# Laparoscopic Cholecystectomy for Gallbladder Calculosis in Fibromyalgia Patients: Impact on Musculoskeletal Pain, Somatic Hyperalgesia and Central Sensitization

**DOI:** 10.1371/journal.pone.0153408

**Published:** 2016-04-15

**Authors:** Raffaele Costantini, Giannapia Affaitati, Francesca Massimini, Claudio Tana, Paolo Innocenti, Maria Adele Giamberardino

**Affiliations:** 1 Institute of Surgical Pathology, “G. D’Annunzio” University of Chieti, Chieti, Italy; 2 Fibromyalgia and Musculoskeletal Pain Center, Department of Medicine and Science of Aging and Ce.S.I., “G. D’Annunzio” University of Chieti, Chieti, Italy; 3 Institute of Clinical Pathology, “G. D’Annunzio” University of Chieti, Chieti, Italy; 4 Internal Medicine Unit, Guastalla Hospital, AUSL Reggio Emilia, Reggio Emilia, Italy; University of Texas at Dallas, UNITED STATES

## Abstract

Fibromyalgia, a chronic syndrome of diffuse musculoskeletal pain and somatic hyperalgesia from central sensitization, is very often comorbid with visceral pain conditions. In fibromyalgia patients with gallbladder calculosis, this study assessed the short and long-term impact of laparoscopic cholecystectomy on fibromyalgia pain symptoms. Fibromyalgia pain (VAS scale) and pain thresholds in tender points and control areas (skin, subcutis and muscle) were evaluated 1week before (basis) and 1week, 1,3,6 and 12months after laparoscopic cholecystectomy in fibromyalgia patients with symptomatic calculosis (n = 31) vs calculosis patients without fibromyalgia (n. 26) and at comparable time points in fibromyalgia patients not undergoing cholecystectomy, with symptomatic (n = 27) and asymptomatic (n = 28) calculosis, and no calculosis (n = 30). At basis, fibromyalgia+symptomatic calculosis patients presented a significant linear correlation between the number of previously experienced biliary colics and fibromyalgia pain (direct) and muscle thresholds (inverse)(p<0.0001). After cholecystectomy, fibromyalgia pain significantly increased and all thresholds significantly decreased at 1week and 1month (1-way ANOVA, p<0.01-p<0.001), the decrease in muscle thresholds correlating linearly with the peak postoperative pain at surgery site (p<0.003-p<0.0001). Fibromyalgia pain and thresholds returned to preoperative values at 3months, then pain significantly decreased and thresholds significantly increased at 6 and 12months (p<0.05-p<0.0001). Over the same 12-month period: in non-fibromyalgia patients undergoing cholecystectomy thresholds did not change; in all other fibromyalgia groups not undergoing cholecystectomy fibromyalgia pain and thresholds remained stable, except in fibromyalgia+symptomatic calculosis at 12months when pain significantly increased and muscle thresholds significantly decreased (p<0.05-p<0.0001). The results of the study show that biliary colics from gallbladder calculosis represent an exacerbating factor for fibromyalgia symptoms and that laparoscopic cholecystectomy produces only a transitory worsening of these symptoms, largely compensated by the long-term improvement/desensitization due to gallbladder removal. This study provides new insights into the role of visceral pain comorbidities and the effects of their treatment on fibromyalgia pain/hypersensitivity.

## Introduction

Fibromyalgia is a chronic pain syndrome affecting 4–7% of the general population, prevalently women [1]. Its diagnostic criteria were first introduced by the American College of Rheumatology (ACR) in 1990 [2]: (1) presence of diffuse musculoskeletal pain of at least 3 months’ duration; (2) positivity of at least 11 out of 18 specific body sites called tender points (TePs), i.e., exquisite tenderness elicited at these points with a standard pressure of 4 kg-f exerted either manually or measured via a pressure algometer. New criteria were recently proposed [3], where tender point evaluation is no longer indispensable and other clinical requirements related to the frequent FMS comorbidities (e.g., visceral pain, headache, sleep and mood disorders) are introduced [4–6]. The new criteria are less restrictive, thus an ACR 1990 diagnosis of FMS is normally confirmed with their application [7]. The pathophysiology of fibromyalgia is still under investigation; knowledge so far acquired, however, indicates that a key role in the syndrome is played by phenomena of central amplification of pain signals, due to an imbalance of neurotransmitters involved in nociceptive transmission/control in the Central Nervous System (CNS), in genetically predisposed individuals [1,8,9]. Central sensitization in FMS is testified by a generalized decrease in pain threshold to different stimuli at somatic level in superficial and deep tissues of the body wall (skin, subcutis and muscle) not only in painful but also in nonpainful areas [1,5,7,10]. In spite of the basically central nature of the syndrome, however, the role of peripheral conditions as possible pain exacerbating factors in FMS has being increasingly recognized in recent years, with nociceptive inputs from the periphery enhancing the level of central sensitization and consequently the extent of pain symptoms/hypersensitivity typical of the syndrome [7,11,12].

Surgery is a powerful source of peripheral impulses, leading to possible sensitization, and fibromyalgia patients have indeed been shown to be at increased risk of experiencing enhanced pain subsequent to various surgical procedures [13–18]. No research, however, has so far been conducted in fibromyalgia patients on the effects of laparoscopic cholecystectomy; this minimally invasive procedure, known to produce significantly lesser consequences than other more invasive surgery in normal subjects [4,16,19–21], represents the gold standard for treatment of gallbladder calculosis, a common occurrence both in the general and FMS population [22]. On this basis, the aim of our study was firstly to assess, in FMS patients with comorbid gallbladder calculosis, the profile of FMS pain, tenderness and general sensitivity to pain stimuli, as compared to FMS without gallbladder calculosis and gallbladder calculosis without fibromyalgia. Secondly, we aimed at evaluating short and long-term effects (up to 1 year) of laparoscopic cholecystectomy specifically on FMS pain symptomatology. In particular, we assessed the spontaneous diffuse musculoskeletal pain, tenderness at tender point sites and pain sensitivity in superficial (skin, subcutis) and deep (muscle) somatic tissues in control areas (outside the TePs) through measurement of pain thresholds to more than one stimulus modality (electrical and pressure stimulation) as compared to the profile of the same parameters over the same time period in FMS patients without and with gallbladder calculosis not undergoing surgery, as well as non-FMS patients with gallbladder calculosis undergoing cholecystectomy [7,12].

## Materials and Methods

### Patients and inclusion criteria

Patients affected with fibromyalgia primarily diagnosed according to the ACR criteria (1990), with confirmation of the diagnosis based on the 2010 revised preliminary criteria, with and without concurrent gallbladder calculosis, were considered [2,3]. Confirmation of the diagnosis was performed based on a retrospective analysis of data present in the patients’ records at the first visit, regarding site/distribution of FMS pain areas and the presence of comorbidities (e.g., headache, sleep and mood disorders, visceral pain]. They were all outpatients referred to the Fibromyalgia and Musculoskeletal Pain Center of the Department of Medicine and Science of Aging of the “G. D’Annunzio” University of Chieti in the period January 2003- December 2013. Patients with gallbladder calculosis not meeting FMS criteria were also considered; these were referred to the Institute of Surgical Pathology of the same University over the same period. The protocol was approved by the Institutional Review Board—Department of Medicine and Science of Aging—“G. D’Annunzio” University of Chieti, and adhered to the principles expressed in the Declaration of Helsinki. A written informed consent was obtained from all patients (see inclusion criteria).

For fibromyalgia, the following groups were considered:

- [FMS+sGb+Cholec]: patients with fibromyalgia + symptomatic gallbladder calculosis, scheduled to undergo laparoscopic cholecystectomy in the forthcoming month;
- [FMS+aGb]: patients with fibromyalgia + asymptomatic gallbladder calculosis, not scheduled for cholecystectomy;
- [FMS]: patients with fibromyalgia without gallbladder calculosis;
- [FMS+sGb]: patients with fibromyalgia + symptomatic gallbladder calculosis, not willing to undergo laparoscopic cholecystectomy in the immediate future.

A further group was considered:

- [sGb+Cholec]: non-fibromyalgic patients + symptomatic gallbladder calculosis scheduled for laparoscopic cholecystectomy in the forthcoming month.

In patients to be subjected to laparoscopic cholecystectomy surgery was performed either at the Institute of Surgical Pathology of the “G. D’Annunzio” University of Chieti or in other Surgery Units of their choice throughout Italy.

Inclusion criteria for [FMS+sGb+Cholec] were: female sex; age 18–70 years; a diagnosis of FMS performed by a specialist 2–5 years previously, with the start of symptoms not earlier than 6 years before, average intensity of diffuse pain ≥50 mm of VAS, on a stable dose of amitriptyline (10 mg/day)–in the preceding 3 months; gallbladder stone diagnosed at least 1 year previously, longest stone diameter 10–40 mm documented at abdominal ultrasounds (AUs), symptomatic for biliary pain episodes (first and last episodes maximum 5 years and 1 month before), respectively; laparoscopic cholecystectomy scheduled in the next month; a negative clinical history for other recurrent/chronic pain conditions (except FMS and symptomatic gallbladder calculosis) over the past 5 years and for any medical condition known to influence general sensitivity to pain (e.g., uncontrolled hypertension, diabetes); exclusion of previous and current conditions known to represent a preoperative risk factor for conversion in laparoscopic cholecystectomy (e.g., ischemic heart disease, uncontrolled arterial hypertension, liver pathologies, chronic obstructive pulmonary disease; severe obesity, diabetes, previous pancreatitis, ultrasound signs of acute cholecystitis, previous upper abdominal surgery); exclusion of major psychiatric disorders at specialistic psychiatric examination; informed, written consent to participate in the study [7,19,22–25].

Inclusion criteria for [FMS+aGb] were the same as for [FMS+sGb+Cholec], except that the gallbladder calculosis had to be asymptomatic with respect to colic pain (calculosis discovered by chance at AUs performed for different reasons within the previous 6 months) and no cholecystectomy was scheduled in the immediate future.

Inclusion criteria for FMS were the same as for [FMS+sGb+Cholec], except that patients had to be free from gallbladder calculosis as documented by AUs performed no more than 6 months previously.

Inclusion criteria for [FMS+sGb] were the same as for [FMS+sGb+Cholec], except that patients had to be unwilling to undergo cholecystectomy in the immediate future.

Inclusion criteria for [sGb+Cholec] were the same as for [FMS+sGb+Cholec], except that a diagnosis of fibromyalgia had to be excluded at a specialistic evaluation.

### Experimental protocol

The study was carried out in two phases of 1-year duration each. The first phase was conducted in all groups. The second phase was conducted only in patients with fibromyalgia + symptomatic gallbladder calculosis not undergoing cholecystectomy during the first year. All evaluations were carried out at the Fibromyalgia and Musculoskeletal Pain Center of Chieti University.

#### Phase 1 –First year of evaluation

In patients scheduled for laparoscopic cholecystectomy, evaluations were carried out at six time points: in basal conditions (1 week before surgery) and 1 week, 1 month, 3, 6 and 12 months after the operation. Patients not undergoing cholecystectomy underwent the same evaluations at comparable time points as those subjected to surgery.

In all FMS patients, the recorded parameters were:

1. average intensity of diffuse musculoskeletal pain from FMS on a Visual Analogue Scale (VAS) (self-described average in a single measurement);
2. pain sensitivity at the 18 TePs through measurement of pressure pain thresholds (PPTs) via Fischer’s algometer (to calculate the mean PPT in all points)[26];
3. pain sensitivity in areas outside the TePs (control areas), i.e., in 3 body sites: trapezius, deltoid and quadriceps of one side, through measurement of electrical (EPTs) and pressure pain thresholds in muscle and of electrical pain thresholds in overlying subcutis and skin [10];
4. number of previously experienced biliary colics (colic defined as: accessional pain in the upper right abdominal quadrant, with or without radiation to the back, towards the inferior angle of the scapula) (only in FMS patients with gallbladder calculosis).

In non-FMS patients, in basal conditions evaluations c-d were performed.

Patients subjected to laparoscopic cholecystectomy were also requested to note, in an ad-hoc diary, the intensity of the pain (VAS scale) perceived at the site of the intervention on a daily basis, during the first post-operative week [27]. Diaries had to be brought back to the experimenters at their first control visit at the Fibromyalgia Center after the operation.

A total of 116 FMS patients meeting the inclusion criteria were included in the study: n = 31 for [FMS+sGb+Cholec] (mean age: 42.3 ± 5.2 SD years), n = .28 for [FMS+aGb] (41.1 ± 5.9 yrs); n = 30 for [FMS](41.5 ± 6.2 yrs) and n = 27 for [FMS+sGb](40 ± 7.6 yrs). A total of 26 non-FMS patients with symptomatic gallbladder calculosis [sGb+Cholec] (39.3 ± 4.8 yrs) meeting the inclusion criteria were included in the study. All groups of patients did not differ in mean age. [FMS+sGb] patients were informed that they were free to interrupt the study protocol should they decide to undergo cholecystectomy at any time during the first year of evaluation.

#### Phase 2—Second year of evaluation

This phase was designed only for the 27 patients [FMS+sGb] who had been unwilling to undergo cholecystectomy at the beginning of the first year. None of them interrupted the study during this period. At the start of the second year, 20 decided to be scheduled for the intervention within the next month [FMS+sGb with delayed Cholec](41.3±7.5 yrs) while 7 of them continued to refuse to undergo cholecystectomy [FMS+sGb without delayed Cholec](38.7±8.2 yrs). All of them accepted to continue to be monitored for their FMS symptoms for a further year, according to the same protocol as for the first year of evaluation.

### Pain threshold measurement to pressure stimulation

A pressure dynamometer was used (Fischer’s algometer, Pain Diagnostic and Treatment, Inc., Great Neck, NY)[26]. The device has a rounded probe 1 cm in diameter, which was placed perpendicularly on each site to evaluate. The pressure was gradually increased by 0.1 kg-f/s until the patient reported a first sensation of discomfort, and the corresponding kg-f value was recorded as the threshold for that site. In all patients PPTs were assessed at all Tender Point Sites (specific FMS painful sites), and in the trapezius, deltoid and quadriceps of one side (control sites). In each of these three muscles, two different points were evaluated (lateral and medial for trapezius—upper and lower for deltoid and quadriceps). For each patient, the mean threshold was calculated of values recorded at the 18 TePs as the reference value for the specific FMS painful sites, and the mean threshold of the 6 values recorded in trapezius, deltoid and quadriceps as the reference value for the control sites.

### Pain threshold measurement to electrical stimulation

A computerized constant current electrical stimulator (R.S.D. Stimulator, prototype, Florence 1997) was used. Delivered stimuli were 18-ms trains of 0.5-ms monophasic square wave pulses, frequency 310 Hz, repeated automatically every 2 s (1 train stimulus every 2 s) through needle or surface electrodes (for deep tissues of the body wall and for skin, respectively). In particular, two monopolar needle electrodes 0.3 mm in diameter, 20 mm in length (Teflon isolation except for 2 mm at the tip) were inserted vertically below the skin surface, 1.5 cm apart. For the muscle, their tips were made to penetrate deep under the fascia; their intramuscular position was verified by observing electrode movement under voluntary contraction and/or low-intensity electrical stimulation of the muscle. For the subcutis these same electrodes were used, pulled out of the muscle fascia at the same sites. Electrodes used to evaluate the skin were a 10-mm diameter circular plate in Ag/AgCl (reference electrode) and a cylinder in Ag/AgCl with a 0.3 mm-diameter base (stimulating electrode), placed 1 cm apart onto the skin surface with interposition of conductor paste. The pressure exerted by the stimulating electrode onto the skin surface was maintained constant through an adjustable spring device connected to the electrode itself (*see* [5,7,22,23,28] for a detailed description of the technique).

The evaluated sites in subctutis/muscle were: the lateral aspect of the upper border of the trapezius (not coinciding with the TeP site, electrodes inserted in the horizontal direction), the lower half of the deltoid and the lowest third of the quadriceps (anterior aspect of the thigh). The stimulation process started at a very low current intensity (0.01 mA), which was then automatically increased with each stimulus repetition by 0.03 mA steps until a first report of a non-painful sensation (typically described as a “slight twitch” for the muscle, a “tingling” sensation for the subcutis, a sensation of “slight touch” for the skin) and then by 0.1 mA steps until a distinct pain sensation was reported (“cramp-like” pain for the muscle, “linearly radiating tingling pain” for the subcutis and “pricking pain” for the skin). The method of the limits was applied to assess the pain threshold. The value corresponding to the first pain perception was stored by the device; the stimulus intensity was then decreased, again by 0.1 mA steps, until pain disappeared and this second value was again stored. The stimulus intensity was then increased again until the sensation re-appeared, with storage of this third value. The device automatically calculated the mean of these three values, which was regarded as the final pain threshold for that specific site and tissue. The patients had to signal the appearance/disappearance of the sensation by pressing a button connected to the stimulator.

Patients were instructed to report the very first sensation of pain and not to bear any pain before reporting it. They were also informed they were free to discontinue the session at any moment without any penalty.

Measurements were always performed at the same time of day (10:00–12:00 a.m.), in the pain-free interval. No acute medication was allowed for 72 hours before the evaluation, to avoid any direct pharmacologic influence on the pain threshold. During the evaluation the patients lay comfortably on an adjustable couch in a quiet room [22]. The researchers evaluating thresholds were not aware of the group the patient belonged to.

### Surgery

Elective laparoscopic cholecystectomy was performed in accordance with standard procedural techniques [27] by expert laparoscopic surgeons, in hospitalized patients (2–4 days), under general anesthesia. Premedication was carried out with paracetamol and non-steroidal anti-inflammatory drugs (NSAIDs); in-hospital postoperative pain was dealt with using opioids (morphine) on demand, while paracetamol and NSAIDs were prescribed on discharge (to be taken by the patients at home if needed)[29].

### Statistical analysis

Means ± Standard Deviation (SD) were calculated for all parameters at each detection time.

A two-way analysis of variance (ANOVA) was applied to assess the effects of time [basis, 1week, 1 month, 3, 6 and 12 months] and clinical condition [i.e., patient groups] for each evaluated parameter during the 1^st^ year study. In each group, the trend for temporal variation of each parameter was evaluated via ANOVA for repeated measures with post-hoc tests for internal comparisons. The comparison among groups at each determination time for each parameter was performed via 1-way ANOVA. For the 2^nd^ year study, the trend for variation of each parameter in time was evaluated via ANOVA for repeated measures. Comparisons of the two groups (Fs+sGb without and with cholecystectomy) in basal conditions was performed via Student’s t test for unpaired samples. In each of these 2 groups, the comparison between basal values of the 1^st^ year and basal values of the 2^nd^ year was carried out via Student’s t-test for paired samples.

The linear regression analysis was employed to evaluate:

1. in patients with symptomatic gallbladder calculosis, the possible correlation between the number of previously experienced biliary colics and FMS parameters in basal conditions (VAS of diffuse pain, pain thresholds at TeP and control areas);
2. in patients undergoing cholecystectomy, the possible correlation between extent of postoperative pain (maximal VAS value at the operation site during the first postoperative week) and the change in FMS parameters postoperatively (i.e., at each postoperative determination time, percentage of increase/decrease with respect to basal values of VAS of diffuse musculoskeletal pain and of thresholds). The level of significance was set at P<0.05.

The softwares used for the statistics were GraphPad InStat and GraphPad Prism. Raw data of the evaluated parameters are reported in S1–S13 Tables.

## Results

### Phase 1—First year of evaluation

At 2-way ANOVA, a significant effect was found of time (basis, 1w, 1m, 3m, 6m, 12m) and clinical condition ([FMS+sGb+Cholec]; [FMS+aGb]; [FMS]; [FMS+sGb]; [sGb+Chol]) for all the evaluated parameters during the 1^st^ year study, in particular:

- for VAS, time (P<0.0001; F: 6.366) and clinical condition (P<0.0001; F: 54.52);
- for PPTs in TePs, time (P<0.0001; F:5.764) and clinical condition (P<0.0001; F: 12.81);
- for EPTs in skin, time (P<0.0001; F: 12.31) and clinical condition (P<0.0001; F: 85.08);
- for EPTs in subcutis, time (P<0.0001; F: 5.266) and clinical condition (P<0.0001; F: 64.44);
- for EPTs in muscle, time (P<0.0001; F: 7.975) and clinical condition (P<0.0001; F: 240.4);
- for PPTs in muscle, time (P<0.0001; F: 7.607) and clinical condition (P<0.0001; F: 528.2).

#### Basal conditions

Biliary symptoms. Patients of the three groups with symptomatic gallbladder calculosis, [FMS+sGb+Cholec], [FMS+sGb] and [sGb+Cholec], did not differ for the number of previously experienced biliary colics (Table 1).

**Table 1:** Biliary and fibromyalgia symptoms in basal conditions (1^st^ year-study).

Patient Group	Biliary colics (n)	FMS pain (mm VAS)	PPTs in TePs (kg-f)
*FMS+sGb+Cholec (n = 31)	3.26 ± 1.63	75.52 ± 3.44^#^^@^	1.26 ± 0.31^@^
#FMS+aGb (n = 28)	---------	65.78 ± 4.86	1.52 ± 0.46
@FMS (n = 30)	---------	67.93 ± 6.71	1.63 ± 0.46
&FMS+sGb (n = 27)	3.07 ± 1.73	76.41 ± 4.53^#^^@^	1.30 ± 0.32^@^
§sGb+Cholec (n = 26)	3.42 ± 1.86	------------	------------

PPTs: pressure pain thresholds; TePs: tender points. Means ± SD. At 1-way ANOVA: no significant trend for biliary colics (P = 0.7655, F = 0.2682); significant trend for VAS (P<0.0001 F = 32.226) and PPTs in TePs (P<0.0008; F = 6.060). The symbols in superscript denote a significant difference with respect to the relative patient group.

* = FMS+sGb+Cholec;

^#^ = FMS+aGb;

^@^ = FMS;

^&^ = FMS+sGb;

^§^ = sGb+Cholec

Fibromyalgia symptoms. Spontaneous fibromyalgia pain (VAS of FMS pain) and pressure pain thresholds at tender point sites (PPTs in TePs) were similar between [FMS+sGb+Cholec] and [FMS+sGb] and between [FMS+aGb] and [FMS], respectively. In fibromyalgia patients with symptomatic gallbladder calculosis with respect to fibromyalgia patients with asymptomatic gallbladder calculosis and without gallbladder pathology: VAS of FMS pain was significantly higher and PPTs in TePs were lower, the difference being significant for FMS+sGb+Cholec and FMS+sGb vs FMS (Table 1).

**Fig 1:**
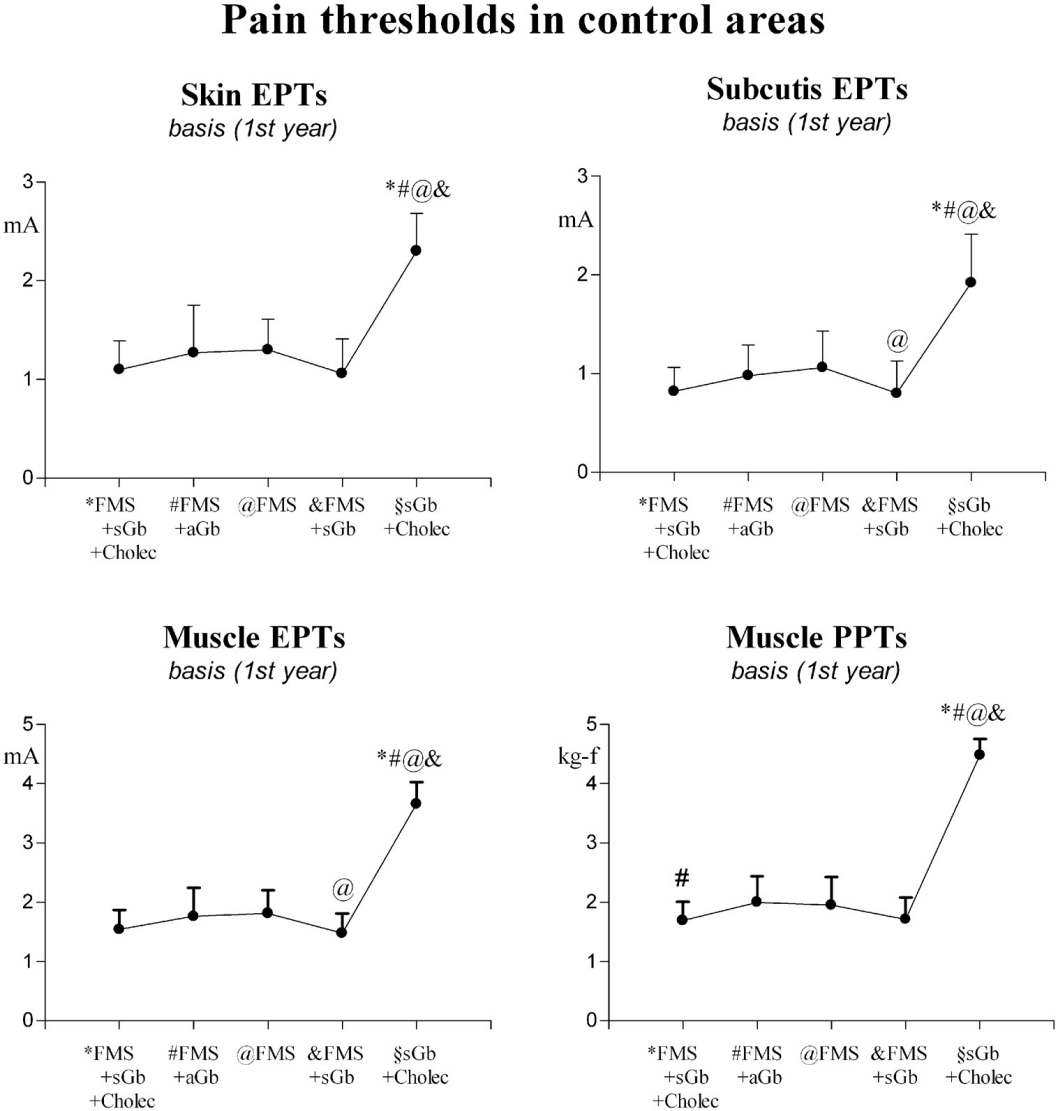
Pain thresholds in control areas in basal conditions (1^st^ year study). Means ± SD for all patients’ groups. Significant trend at 1-way ANOVA: P<0.0001 for all parameters (F = 52.251 for EPTs in skin, 45.450 for EPTs in subcutis, 149.21 for EPTs in muscle, 254.60 for PPTs in muscle). The symbols over SD bars denote a significant difference with respect to the other groups.

Pain sensitivity in control sites. All thresholds were similar between [FMS+sGb+Cholec] and [FMS+sGb] and between [FMS+aGb] and [FMS]. They were lower in the two fibromyalgia groups with symptomatic gallbladder calculosis, [FMS+sGb+Cholec] and [FMS+sGb], than in the two other fibomyalgia groups, [FMS+aGb] and [FMS]. Differences were significant: for electrical subcutis and muscle thresholds (lower in [FMS+sGb] than in FMS), for pressure muscle thresholds (lower in [FMS+sGb+Cholec] than [FMS+aGb]). All thresholds in the four fibromyalgia groups were significantly lower than those in the non-fibromyalgia group with symptomatic gallbladder calculosis (Fig 1).

**Fig 2:**
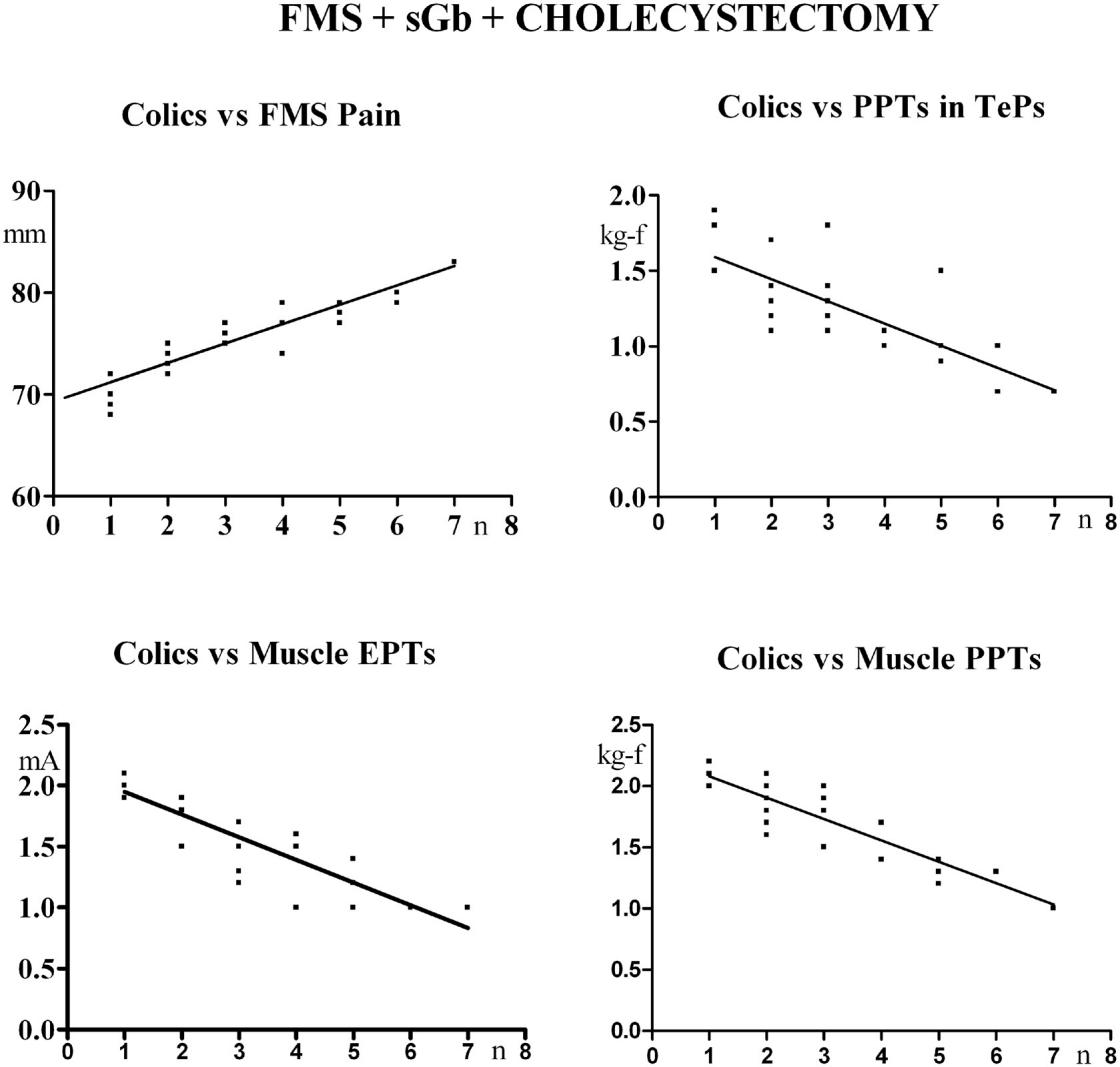
Patients [FMS+sGb+Cholec] (n = 31). Linear correlations between number of previously experienced colics and FMS pain, pain thresholds in TePs, electrical and pressure muscle pain thresholds in control areas in basal conditions.

Biliary symptoms vs fibromyalgia symptoms / pain sensitivity. In [FMS+sGb+Cholec], a significant direct linear correlation was present between the number of previously experienced colics and the VAS for FMS pain [Y = 1.9X + 69.326; (r) = 0.9006; P< 0.0001] and a significant inverse linear correlation was found between the number of colics and pressure pain thresholds in TePs [Y = -0.1472 X+ 1.738; (r) = -0.768; P < 0.0001], electrical muscle pain thresholds [Y = -0.1856X + 2.134; (r) = -0.8837; P < 0.0001] and pressure muscle pain thresholds [Y = -0.1751X + 2.258; (r) = -0.8899; P < 0.0001] in control areas (Fig 2).

There was no significant correlation between number of previously experienced colics and electrical pain thresholds in skin [P = 0.5155; (r) = 0.1214] and subcutis [P = 0.2071; (r) = -0.2330].

**Fig 3:**
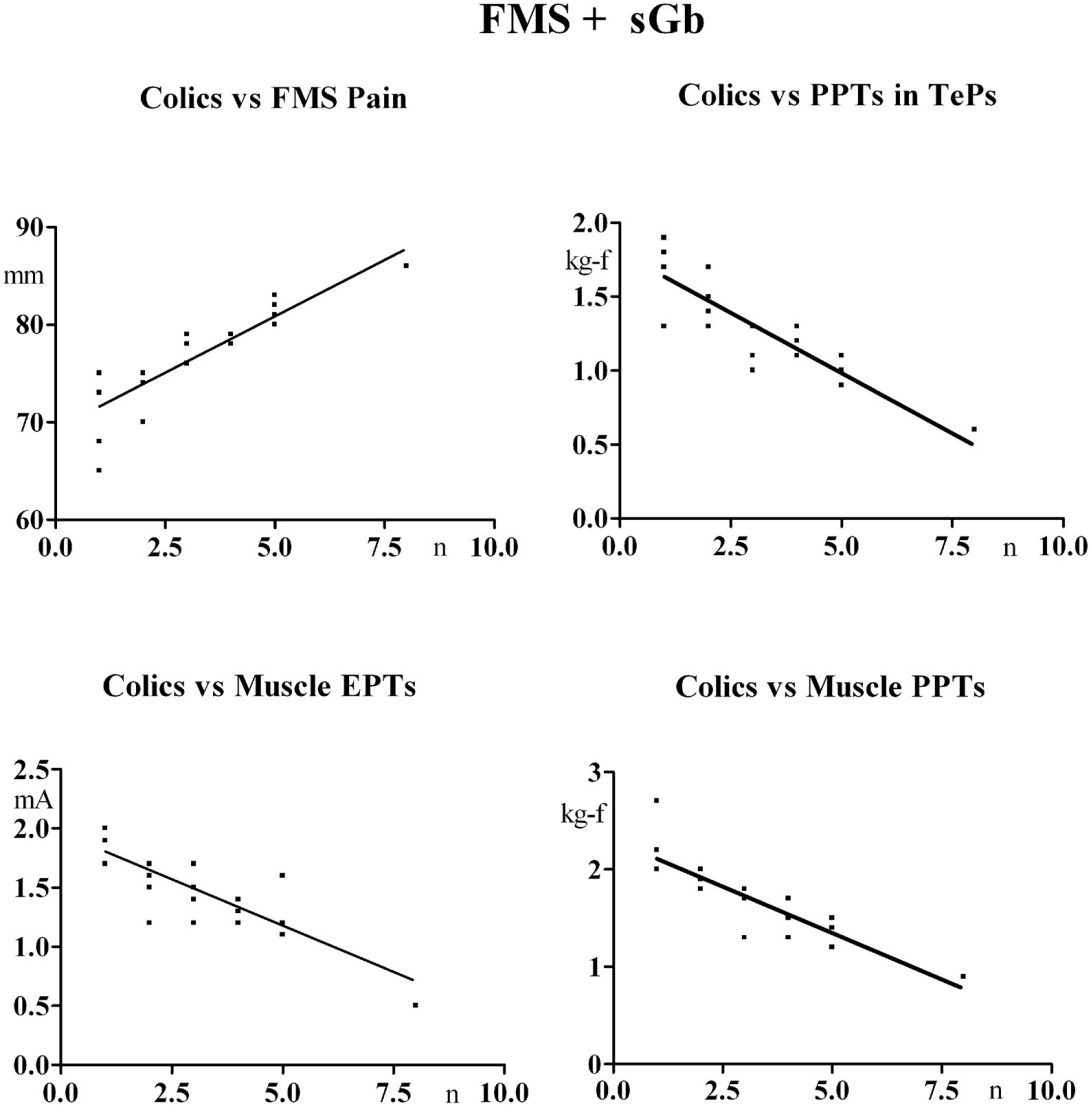
Patients [FMS+sGb] (n = 27). Linear correlations between number of previously experienced colics and FMS pain, pain thresholds in TePs, electrical and pressure muscle pain thresholds in control areas in basal conditions.

In [FMS+sGb], the number of previously experienced colics had a significant direct linear correlation with the VAS of fibromyalgia pain [Y = 2.314X + 69.293; (r) = 0.8849; P< 0.0001] and an inverse linear correlation with pressure pain thresholds in TePs [Y = -0.1630X + 1.797; (r) = -0.8837; P< 0.0001], electrical muscle pain thresholds [Y = -0.1562X + 1.962; (r) = -0.8208; P < 0.0001] and pressure muscle pain thresholds [Y = -0.1905X + 12.076; (r) = -0.8841; P < 0.0001] in control areas (Fig 3).

There was no significant correlation between the number of previously experienced colics and electrical thresholds in skin [P = 0.0881; (r) = -0.3346) and subcutis (P = 0.4582; (r) = -0.1490].

#### Postoperative pain

All patients scheduled for cholecystectomy underwent surgery without complications. The laparoscopic procedure was carried out as planned in all of them, with no need for conversion.

**Fig 4:**
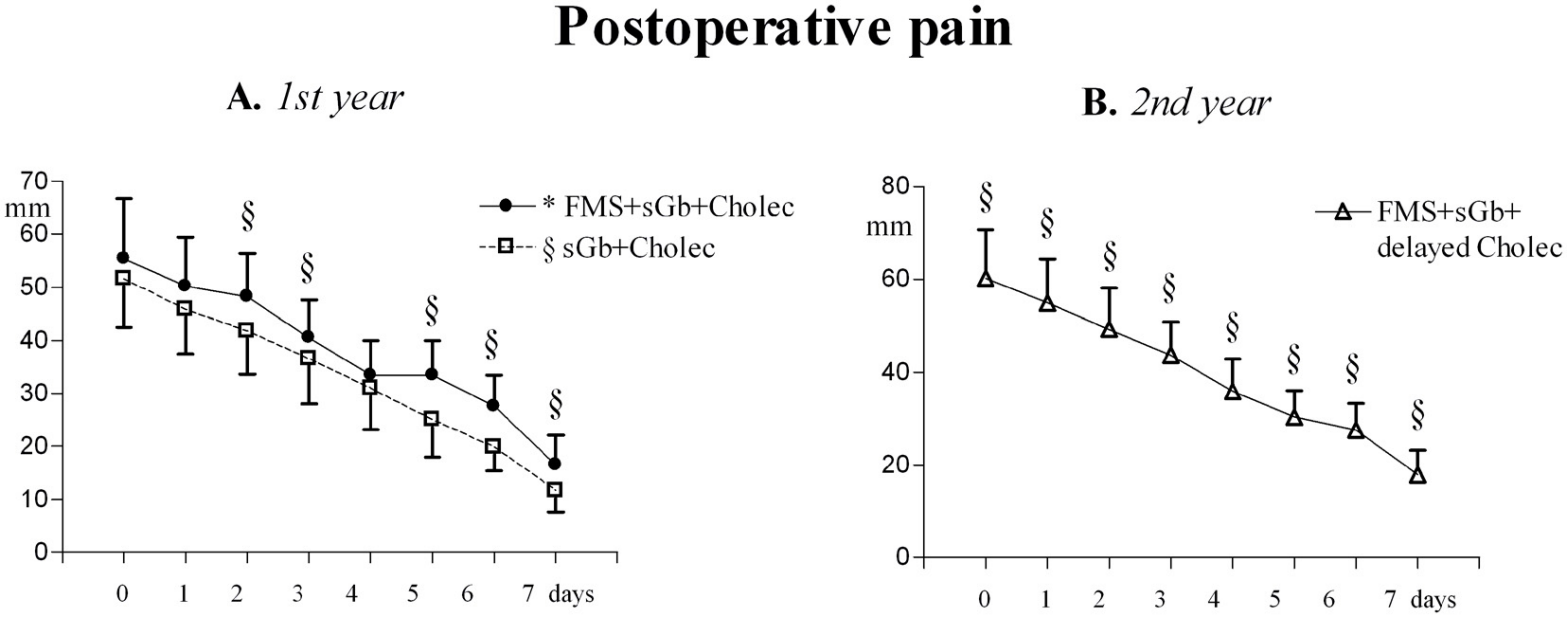
VAS scores of abdominal pain during the first week after laparoscopic cholecystectomy (performed on Day0). Means ± SD. A: 1^st^ year study. B: 2^nd^ year study. Mann-Whitney test for comparison of fibromyalgia groups ([FMS+sGb+Cholec and sGb+Cholec] with the non-fibromyalgia group [sGb+Cholec]). § = significant difference.

VAS scores for pain perceived at abdominal surgery site in the postoperative period (1 week after surgery) are reported in Fig 4A. They were higher in fibromyalgia than non-fibromyalgia patients at all determination times, the difference being significant at days 2,3,5,6 and 7.

#### Evolution of pain parameters during the first year

Biliary symptoms. In patients subjected to cholecystectomy, both fibromyalgic and non-fibromyalgic ([FMS+sGb+Cholec] and [sGb+Cholec]), biliary pain was no longer experienced over the whole year of evaluation after surgery. In fibromyalgia patients with asymptomatic gallbladder calculosis not undergoing cholecystectomy [FMS+aGb], calculosis continued to remain silent with respect to biliary pain throughout the same period. In most fibromyalgia patients with symptomatic gallbladder calculosis who had refused to undergo surgery during the first year, further biliary colics instead occurred over this period (see section: Phase 2 –Second year of evaluation).

**Fig 5:**
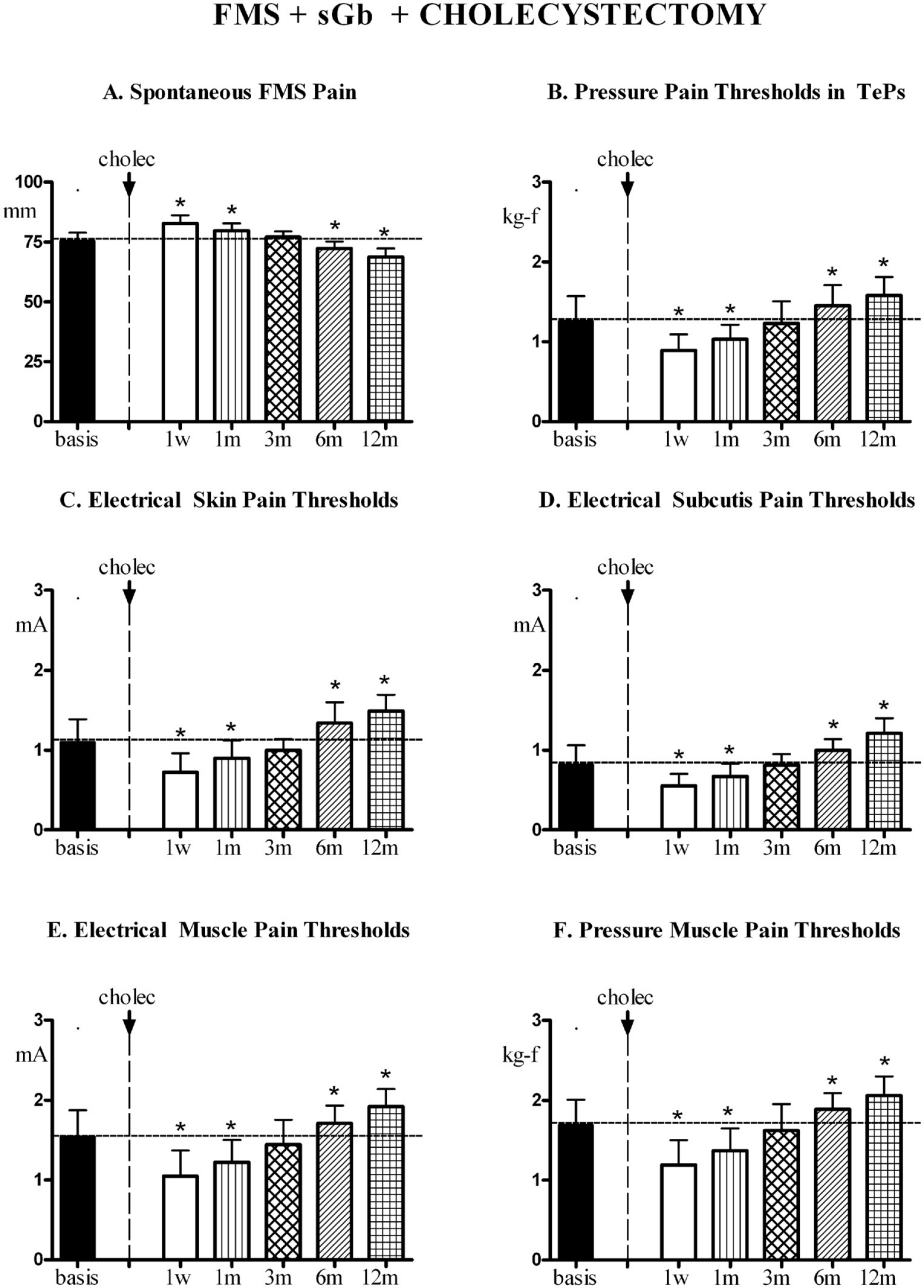
FMS symptoms and pain sensitivity in [FMS+sGb+Cholec]. Patients with fibromyalgia (FMS) plus symptomatic gallbladder calculosis (sGb) subjected to cholecystectomy (cholec) during the first year (n. 31, Means ± SD). (A) Spontaneous fibromyalgia pain (VAS); (B) Pain thresholds to pressure stimulation at the 18 Tender Points (TePs); (C),(D),(E) Pain thresholds to electrical stimulation in skin, subcutis and muscle and to (F) pressure stimulation in muscle in control areas (mean of values recorded in trapezius, deltoid and quadriceps). Basis = pre-operative values; 1w, 1m, 3m, 6m, 12m = 1 week, 1 month, 3 months, 6 months and 12 months after cholecystectomy. ANOVA for repeated measures: significant trend, P<0.0001, for all parameters [F = 104.79 for VAS; F = 58.793 for PPTs in TePs; F = 87.673 for skin EPTs, F = 108.48 for subcutis EPTs; F = 132.79 for muscle EPTs; F = 68.439 for muscle PPTs]. In each graph, asterisks over SD bars denote a significant difference with respect to pre-operative values.

Fibromyalgia symptoms and pain sensitivity in control areas. In fibromyalgia pain subjected to cholecystectomy, [FMS+sGb+Cholec], the evolution of FMS symptoms and pain sensitivity in control areas over the first year of evaluation is reported in Fig 5.

A significant trend for variation was present for all parameters. FMS pain intensity significantly increased with respect to basal values at 1 week and 1 month after the intervention, to return to pre-intervention values at 3 months; it then significantly decreased at 6 and 12 months (A). All thresholds (B-F) significantly decreased at 1 week and 1 month compared to basal values, returned to basal values at 3 months and then significantly and progressively increased at 6 and 12 months.

**Fig 6:**
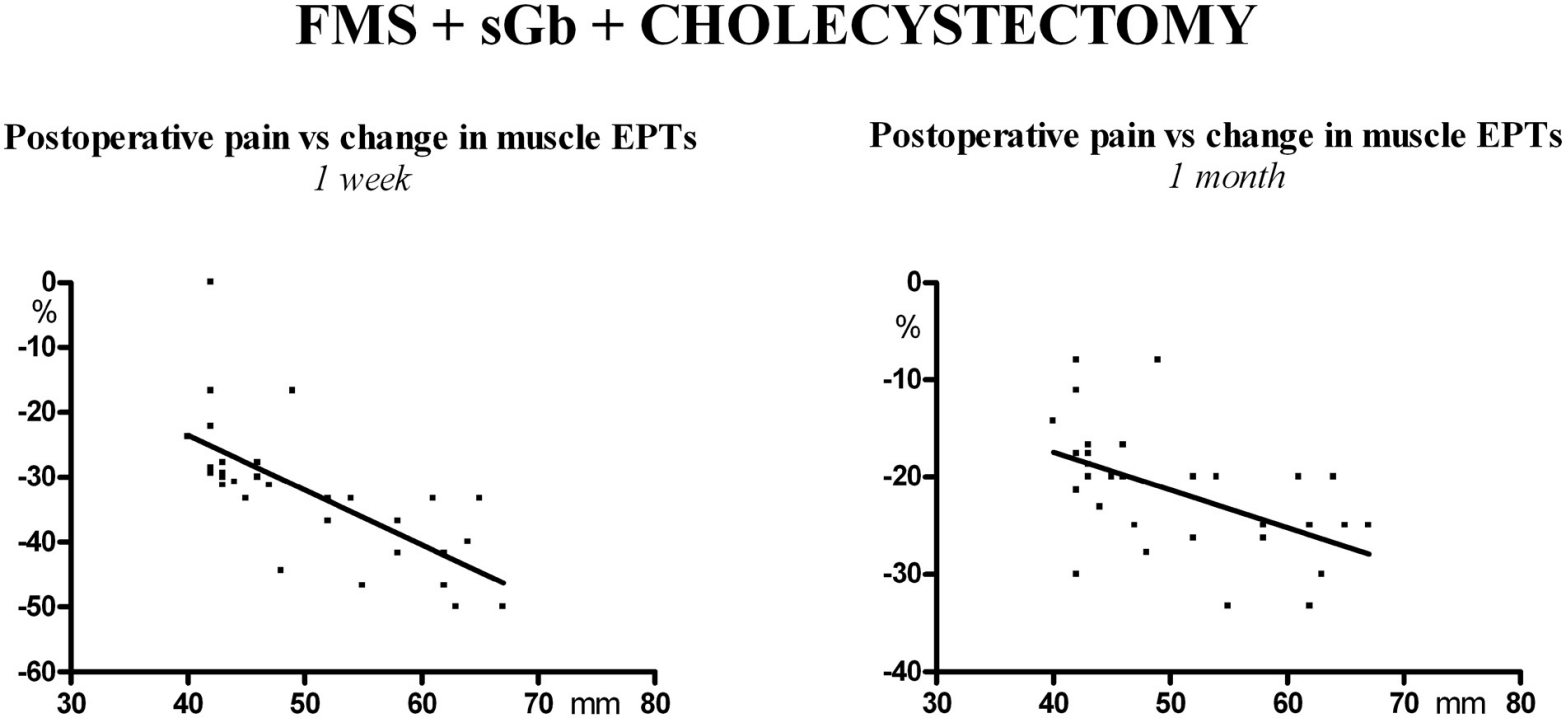
Patients [FMS+sGb+Cholec] (n. 31). Linear correlations between peak postoperative pain and change (decrease) in electrical pain thresholds (EPTs) in muscle of control areas at 1 week and 1 month post-cholecystectomy.

A significant direct linear correlation was found between the peak values of postoperative pain and the change (decrease) in electrical pain thresholds in muscles at 1 week [Y = -0.84X + 10.20; (r) = -0.6950; P< 0.0001] and 1 month [Y = -0.39X -2.00; (r) = -0.5220; P<0.003] (Fig 6).

All other correlations were not significant, as reported below.

Postoperative pain vs change in FMS pain: at 1 week (P = 0.9200; (r) = 0.01880), at 1 month (P = 0.4626; (r) = 0.1369), at 3 months (P = 0.3781; (r) = 0.1640), at 6 months (P = 0.3393; (r) = 0.1775), at 12 months (P = 0.8330, (r) = 0.03948).

Postoperative pain vs change in PPTs in TePs: at 1 week (P = 0.8859; (r) = -0.02687), at 1 month (P = 0.8529; (r) = -0.03473), at 3 months (P = 0.7516; (r) = 0.05924), at 6 months (P = 0.5556; (r) = 0.1101), at 12 months (P = 0.9034; (r) = -0.02272).

Postoperative pain vs change in skin EPTs: at 1 week (P = 0.3949; (r) = -0.1583), at 1 month (P = 0.3408; (r) = 0.1770), at 3 months (P = 0.1046, (r) = 0.2971), at 6 months (P = 0.2977; (r) = 0.1932), at 12 months (P = 0.3513; (r) = 0.1732).

Postoperative pain vs change in subcutis EPTs: at 1 week (P = 0.0552; (r) = -0.3478, at 1 month (P = 0.3704; (r) = -0.1666), at 3 months (P = 0.6703; (r) = -0.07961), at 6 months (P = 0.6800; (r) = -0.07713), at 12 months (P = 0.1249; (r) = -0.2816).

Postoperative pain vs change in muscle EPTs: at 3 months (P = 0.1709; (r) = 0.2523), at 6 months (P = 0.8145; (r) = -0.04394), at 12 months (P = 0.7104; (r) = 0.06947).

Postoperative pain vs change in muscle PPTs: at 1 week (P = 0.6872; (r) = 0.07531), at 1 month (P = 0.6087, (r) = -0.09566), at 3 months (P = 0.8569; (r) = 0.03377), at 6 months (P = 0.3499; (r) = 0.1737), at 12 months (P = 0.9553; (r) = -0.01051).

**Fig 7:**
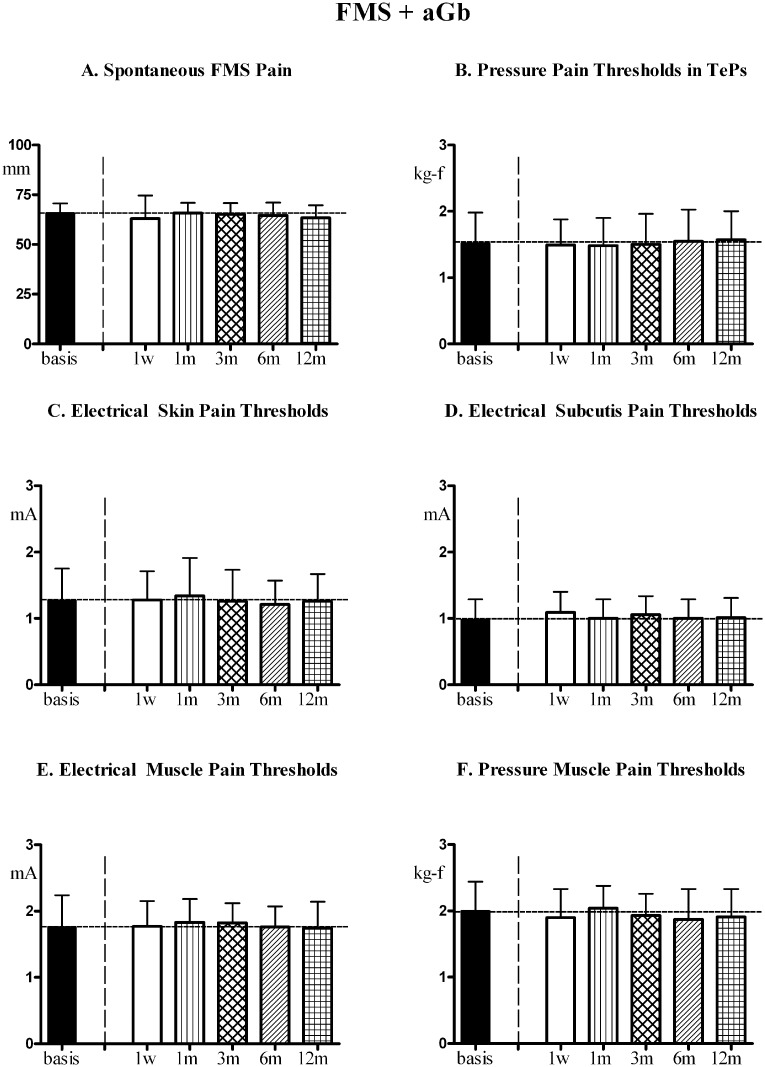
FMS symptoms and pain sensitivity in [FMS+aGb]. Patients with fibromyalgia (FMS) plus asymptomatic gallbladder calculosis (aGb) not subjected to cholecystectomy (n = 28, Means ± SD), followed for a period of 1 year at comparable time points as patients in Fig 5. (A) Spontaneous fibromyalgia pain (VAS); (B) Pain thresholds to pressure stimulation at the 18 Tender Points (TePs); (C),(D),(E) Pain thresholds to electrical stimulation in skin, subcutis and muscle and to (F) pressure stimulation in muscle in control areas (mean of values recorded in trapezius, deltoid and quadriceps). No significant trend for all parameters. ANOVA for repeated measures: for VAS [P = 0.2250; F = 1.409]; for PPTs in TePs [P = 0.8704; F = 0.3670]; for EPTs in skin [P = 0.5311; F = 0.8291]; for EPTs in subcutis [P = 0.2357; F = 1.380]; for EPTs in muscle [P = 0.6849, F = 0.6198], for PPTs in muscle [P = 0.3933, F = 1.046].

**Fig 8:**
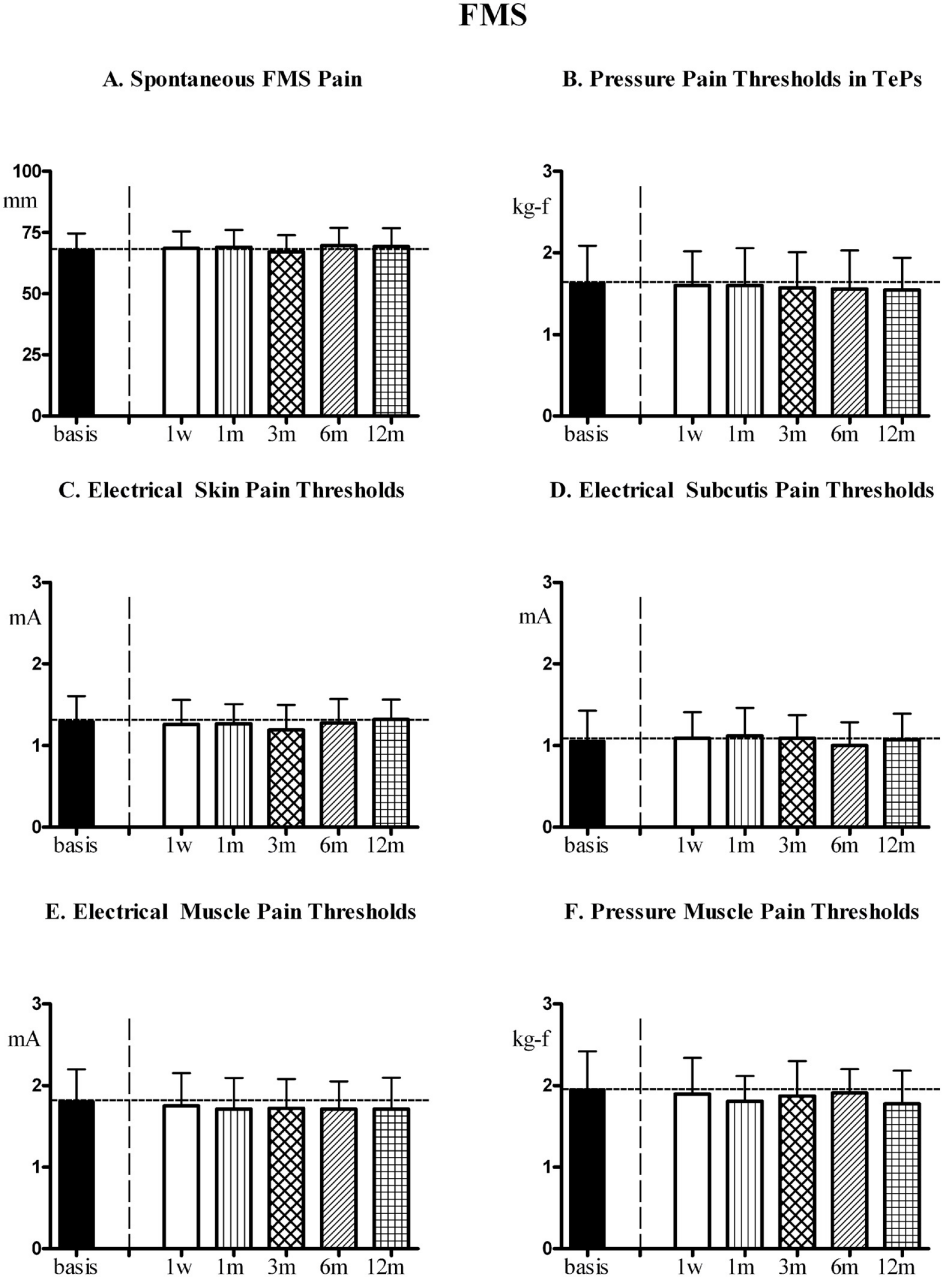
FMS symptoms and pain sensitivity in [FMS]. Patients with fibromyalgia (FMS) without gallbladder calculosis (n = 30, Means ± SD), followed for a period of 1 year at comparable time points as patients in Fig 5. (A) Spontaneous fibromyalgia pain (VAS); (B) Pain thresholds to pressure stimulation at the 18 Tender Points (TePs); (C),(D),(E) Pain thresholds to electrical stimulation in skin, subcutis and muscle and to (F) pressure stimulation in muscle in control areas (mean of values recorded in trapezius, deltoid and quadriceps). No significant trend for all parameters. ANOVA for repeated measures: for VAS [P = 0.1200; F = 1.782], for PPTs in TePs [P = 0.8699; F = 0.3679]; for EPTs in skin [P = 0.1460; F = 1.668], for EPTs in subcutis [P = 0.2237; F = 1.411], for EPTs in muscle [P = 0.1109; F = 1.828], for PPTs in muscle [P = 0.1113; F = 1.826].

In fibromyalgia patients with asymptomatic gallbladder calculosis not subjected to cholecystectomy [FMS+aGb] and fibromyalgia patients without gallbladder calculosis [FMS] the 1st-year evolution of FMS symptoms and pain sensitivity in control areas (at time points comparable to patients undergoing surgery) is reported in Figs 7 and 8, respectively. No significant trend for variation was found for any of the evaluated parameters for both groups.

**Fig 9:**
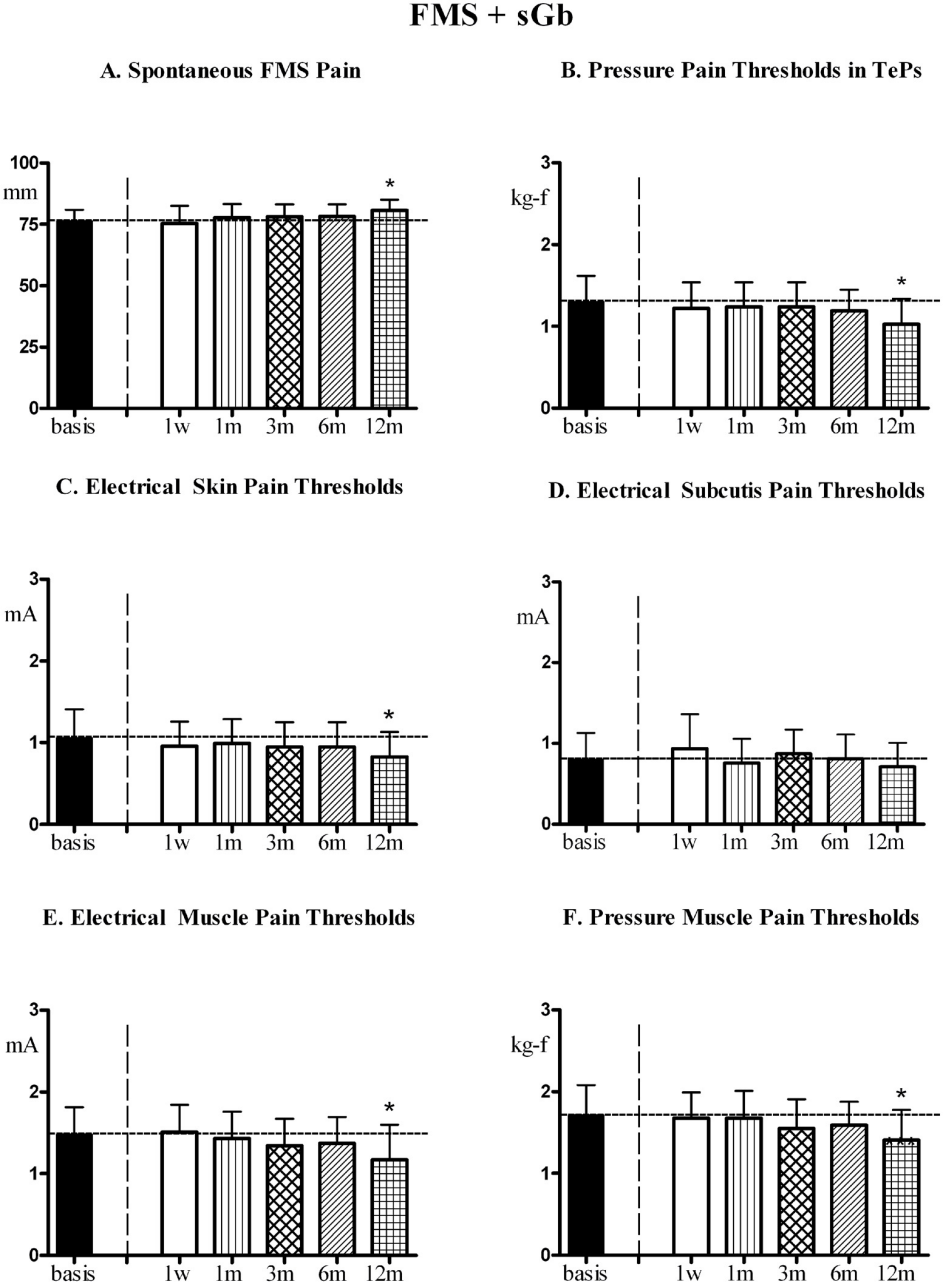
FMS symptoms and pain sensitivity in [FMS+sGb]. Patients with fibromyalgia (FMS) plus symptomatic gallbladder calculosis (sGb) not subjected to cholecystectomy (n = 27, Means ± SD), followed for a period of 1 year at comparable time points as patients in Fig 5. (A) Spontaneous fibromyalgia pain (VAS); (B) Pain thresholds to pressure stimulation at the 18 Tender Points (TePs); (C),(D),(E) Pain thresholds to electrical stimulation in skin, subcutis and muscle and to (F) pressure stimulation in muscle in control areas (mean of values recorded in trapezius, deltoid and quadriceps). ANOVA for repeated measures: for VAS [P<0.0001; F = 6.203]; for PPTs in TePs [P<0.0006, F = 4.797]; for EPTs in skin [P<0.02, F = 3.155], for EPTs in subcutis [P<0.003; F = 3.916]; for EPTs in muscle [P < 0.0001; F = 12.869], for PPTs in muscle [P < 0.0001; F = 7.530].

In fibromyalgia patients with symptomatic gallbladder calculosis not undergoing cholecystectomy during the first year [FMS+sGb], the evolution in time of FMS symptoms and pain sensitivity in control areas over the same period is reported in Fig 9. A significant trend for variation was present for all parameters. Pain intensity slightly increased over time, the increase becoming significant at 12 months with respect to basal values. Pressure pain thresholds in TePs and electrical and pressure muscle pain thresholds in control areas gradually decreased over time, to become significantly lower than in basal conditions at 12 months in all cases except subcutis thresholds.

**Fig 10:**
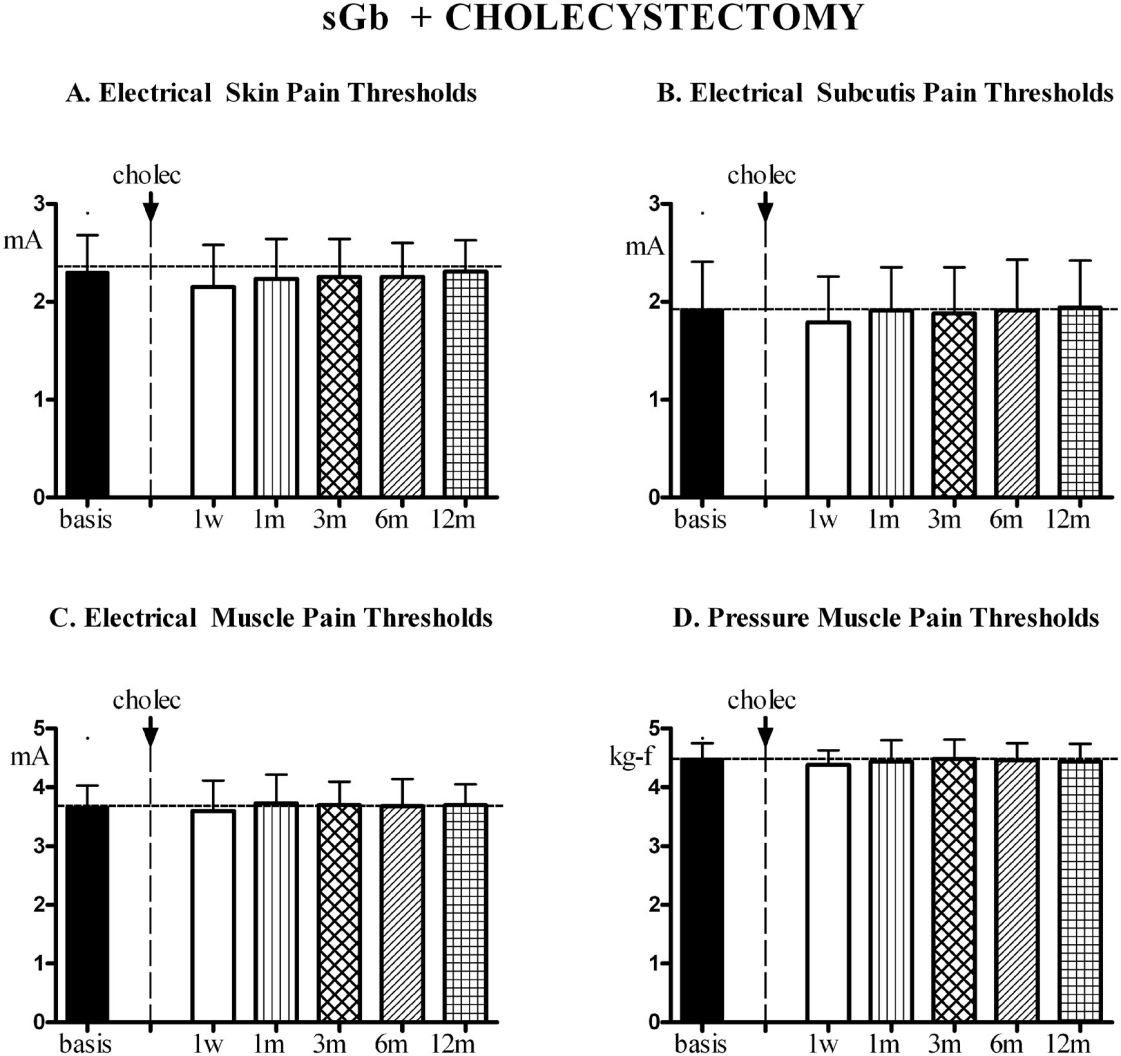
FMS symptoms and pain sensitivity in [sGb+Cholec]. Patients with symptomatic gallbladder calculosis (sGb) subjected to cholecystectomy (Cholec) during the 1^st^ year (n = 26, Means ± SD). Evaluations performed at comparable time points as for patients in Fig 5. (A),(B),(C) Pain thresholds to electrical stimulation in skin, subcutis and muscle and to (D) pressure stimulation in muscle in control areas (mean of values recorded in trapezius, deltoid and quadriceps). No trend for variation in any of the recorded parameters. ANOVA for repeated measures: for EPTs in skin [P = 0.1050; F = 1.866], for EPTs in subcutis [P = 0.2035; F = 1.472]; for EPTs in muscle [P = 0.3216; F = 1.182]; for PPTs in muscle [P = 0.3018; F = 1.224].

In non-fibromyalgic patients with symptomatic gallbladder calculosis undergoing cholecystectomy [sGb+Cholec] the evolution in time of pain thresholds in control areas during the whole year of study is reported in Fig 10. No significant trend for variation was found in any of the evaluated parameters, thresholds remaining stable throughout.

#### Comparison among groups at times 1w-12m

The comparison among different groups at each time point subsequent to basal evaluation, performed via 1-way ANOVA, is reported in Table 2 for Fibromyalgia Symptoms and Table 3 for thresholds.

**Table 2:** Fibromyalgia symptoms. Comparison among groups at times 1w-12m – 1^st^ year study.

Patient Group	1w	1m	3m	6m	12m
*FMS+sGb+Cholec (n = 31)	**VAS** #,@,&	**VAS** #,@	**VAS** #,@	**VAS** #,&	**VAS** #,&
	**PPTs in TePs** #,@,&	**PPTs in TePs** #,@	**PPTs in TePs** #,@	**PPTs in TePs —**	**PPTs in TePs** &
#FMS+aGb (n = 28)	**VAS** *,@,&	**VAS** *,&	**VAS** *,&	**VAS** *,@,&	**VAS** *,@,&
	**PPTs in TePs** *,&	**PPTs in TePs** *	**PPTs in TePs** *	**PPTs in TePs** &	**PPTs in TePs** &
@FMS (n = 30)	**VAS** *, #,&	**VAS** *,&	**VAS** *,&	**VAS** #,&	**VAS** #,&
	**PPTs in TePs** *,&	**PPTs in TePs** *,&	**PPTs in TePs** *,&	**PPTs in TePs** &	**PPTs in TePs** &
&FMS+sGb (n = 27)	**VAS** *,#,@	**VAS** @	**VAS** #,@	**VAS** *,#,@	**VAS** *,#,@
	**PPTs in TePs** *,#,@	**PPTs in TePs** @	**PPTs in TePs** @	**PPTs in TePs** #,@	**PPTs in TePs** *,#,@

1-way ANOVA for VAS: 1 week (P < 0.0001, F = 42.360), 1 month (P < 0.0001, F = 44.103), 3 months (P < 0.0001, F = 46.336), 6 months (P < 0.0001, F = 28.722), 12 months (P < 0.0001, F = 45.542). 1-way ANOVA for PPTs in TePs: 1 week (P < 0.0001, F = 25.766), 1 month (P < 0.0001, F = 15.102), 3 months (P < 0.0007, F = 6.260), 6 months (P < 0.01, F = 4.024), 12 months (P < 0.0001, F = 8.476.

* = FMS+sGb+Cholec;

^#^ = FMS+aGb;

^@^ = FMS;

^&^ = FMS+sGb.

**Table 3:** Pain thresholds in control areas. Comparison among groups at times 1w-12m—1^st^ year study.

Patient Group	1w	1m	3m	6m	12m
*FMS+sGb+ Cholec (n = 31)	**Skin EPTs NS**	**Skin EPTs** #,@,&	**Skin EPTs** #,§	**Skin EPTs** &,§	**Skin EPTs** #,&,§
	**Subcutis EPTs** §	**Subcutis EPTs** #,@,§	**Subcutis EPTs** #,@,§	**Subcutis EPTs** §	**Subcutis EPTs** &,§
	**Muscle EPTs** #,@,&,§	**Muscle EPTs** #,@,§	**Muscle EPTs** #,@,§	**Muscle EPTs** #,@,§	**Muscle EPTs** &,§
	**Muscle PPTs** #,@,&,§	**Muscle PPTs** #,&@,§	**Muscle PPTs** #,§	**Muscle PPTs** &,§	**Muscle PPTs** @,&,§
#FMS+aGb (n = 28)	**Skin EPTs** &,§	**Skin EPTs** *,&,§	**Skin EPTs** *,&,§	**Skin EPTs** &,§	**Skin EPTs** *,&,§
	**Subcutis EPTs** &	**Subcutis EPTs** *,§	**Subcutis EPTs** *,§	**Subcutis EPTs** §	**Subcutis EPTs** &,§
	**Muscle EPTs** *, §	**Muscle EPTs** *,&,§	**Muscle EPTs** *,&,§	**Muscle EPTs** *,&,§	**Muscle EPTs** &,§
	**Muscle PPTs** *,§	**Muscle PPTs** *, &,§	**Muscle PPTs** *,&,§	**Muscle PPTs** &,§	**Muscle PPTs** &,§
@FMS (n = 30)	**Skin EPTs** &,§	**Skin EPTs** *,&,§	**Skin EPTs** §	**Skin EPTs** &,§	**Skin EPTs** &,§
	**Subcutis EPTs** &,§	**Subcutis EPTs** *,&,§	**Subcutis EPTs** *,§	**Subcutis EPTs** §	**Subcutis EPTs** &,§
	**Muscle EPTs** *,§	**Muscle EPTs** *,&,§	**Muscle EPTs** *,&,§	**Muscle EPTs** *,&,§	**Muscle EPTs** &,§
	**Muscle PPTs** *,§	**Muscle PPTs** *,§	**Muscle PPTs** &,§	**Muscle PPTs** &,§	**Muscle PPTs** *,&,§
&FMS+sGb (n = 27)	**Skin EPTs** #,@,§	**Skin EPTs** #,@,§	**Skin EPTs** #,§	**Skin EPTs** *,#,@,§	**Skin EPTs** *,#,@,§
	**Subcutis EPTs** @,§	**Subcutis EPTs** @,§	**Subcutis EPTs** §	**Subcutis EPTs** §	**Subcutis EPTs** #,@,§
	**Muscle EPTs** *,§	**Muscle EPTs** #,@,§	**Muscle EPTs** #,@,§	**Muscle EPTs** #,@,§	**Muscle EPTs** *,#,@,§
	**Muscle PPTs** *,§	**Muscle PPTs** *,#,§	**Muscle PPTs** #,@,§	**Muscle PPTs** *,#,@,§	**Muscle PPTs** *,#,@,§
§sGb+Cholec (n = 26)	**Skin EPTs** #,@,&	**Skin EPTs** *,@,&	**Skin EPTs** *,#,@&	**Skin EPTs** *,#,@,&	**Skin EPTs** *,#,@,&
	**Subcutis EPTs** *,#,@,&	**Subcutis EPTs** *,#,@,&	**Subcutis EPTs** *,#,@,&	**Subcutis EPTs** *,#,@,&	**Subcutis EPTs** *,#,@,&
	**Muscle EPTs** *,#,@,&	**Muscle EPTs** *,#,@,&	**Muscle EPTs** *,#,@,&	**Muscle EPTs** *,#,@,&	**Muscle EPTs** *,#,@,&
	**Muscle PPTs** *,#,@,&	**Muscle PPTs** *,#,@,&	**Muscle PPTs** *,#,@,&	**Muscle PPTs** *,#,@,&	**Muscle PPTs** *,#,@,&

1w,1m,3m,6,12m: 1 week, 1 month, 3,6, and 12 months time points. EPTs: electrical pain thresholds, PPTs: pressure pain thresholds, TePs: tender points.

The symbols under each variable at each time point denote a significant difference with respect to the relative patient group.

* = FMS+sGb+Cholec;

^#^ = FMS+aGb;

^@^ = FMS;

^&^ = FMS+sGb;

^§^ = sGb+Cholec.

1-way ANOVA for skin EPTs: 1 week (P < 0.0001; F = 67.544), 1 month (P < 0.0001; F = 53.124), 3 months (P < 0.0001; F = 64.373), 6 months (P < 0.0001; F = 62.969), 12 months (P < 0.0001; F = 86.983)

1-way ANOVA for subcutis EPTs: 1 week (P < 0.0001; F = 46.493), 1 month (P < 0.0001; F = 65.155), 3 months (P < 0.0001; F = 52.377), 6 months (P < 0.0001; F = 45.750), 12 months (P < 0.0001; F = 49.664)

1-way ANOVA for muscle EPTs: 1 week (P < 0.0001; F = 162.53), 1 month (P < 0.0001; F = 198.43), 3 months (P < 0.0001; F = 210.35), 6 months (P < 0.0001; F = 204.41), 12 months (P < 0.0001; F = 185.02)

1-way ANOVA for muscle PPTs: 1 week (P < 0.0001; F = 326.45), 1 month ((P < 0.0001; F = 387.76), 3 months (P < 0.0001; F = 307.72), 6 months (P < 0.0001; F = 368.96), 12 months (P < 0.0001; F = 307.52).

Of particular note is the comparison at 12 month between fibromyalgia patients with comorbid symptomatic calculosis subjected to cholecystectomy during the 1^st^ year [FMS+sGb+Cholec] and fibromyalgia patients with comorbid symptomatic calculosis who did not undergo the intervention in this 1-year period [FMS+sGb]. Values of FMS pain were significantly lower and those of all thresholds significantly higher in the former vs the latter suggesting that an earlier intervention on the gallbladder more effectively improved FMS spontaneous and evoked symptoms.

### Phase 2—Second year of evaluation

#### Basal conditions

Biliary symptoms. In basal conditions at the beginning of the second year of evaluation, the number of previously experienced colics in the 20 patients who decided to undergo cholecystectomy [FMS+sGb with delayed Cholec] had passed from 3.25 ± 1.80 to 3.65 ± 1.75 (significant increase: P<0.02), while in the 7 patients who continued to refuse cholecystectomy [FMS+sGb without delayed Cholec] it had passed from 2.57 ± 1.51 to 2.86 ± 1.68 (difference not significant, only 1 patient had reported a further colic in the second year).

Fibromyalgia symptoms and pain sensitivity in control areas. Since the second year of evaluation initiated immediately after completion of the first year study, values of FMS symptoms and of pain thresholds recorded at 12 months of the first year were used as basal values for the second year.

In [FMS+sGb with delayed Cholec], VAS scores had passed from 75.9 ± 4.1 to 80.4 ± 4.2 mm (P<0.0007), PPTs in TePs from 1.32 ± 0.32 to 1.09 ± 0.33 kg-f (P<0.05), EPTs in skin from 1.05 ± 0.34 to 0.80± 0.34 mA (P<0.03), EPTs in subcutis from 0.87± 0.34 to 0.92± 0.28 mA (NS, P = 0.66), EPTs in muscle from 1.45±0.36 to 1.22 ± 0.39 mA (NS, P = 0.05), PPTs in muscle from 1.69 ±0.41 to 1.46 ± 0.41 kg-f (P<0.009).

In [FMS+sGb without delayed Cholec] VAS scores had passed from 77.86 ± 5.7 to 81.43 ± 5.1 mm (NS, P = 0.18) PPTs in TePs from 1.21 ± 0.31 to 0.86 ± 0.18 kg-f (P<0.02), EPTs in skin from 1.08 ± 0.39 to 0.84 ± 0.26 mA (NS, P = 0.28), EPTs in subcutis from 0.58 ± 0.13 to 0.98 to 0.31 mA (P<0.02), EPTs in muscle from 1.57± 0.22 to 1.13± 0.18 mA (P<0.02), PPTs in muscle from 1.78 ± 0.25 to 1.5 to 0.33 kg-f (NS, P = 0.11)

The comparison between the two groups in basal conditions of the 1^st^ year showed: for VAS, P = 0.33; for PPTs in TePs, P = 0.44; for EPTs in skin, P = 0.82; for EPTs in subcutis, P<0.05; for EPTs in muscle, P = 0.44; for PPTs in muscle, P = 0.57.

The comparison between the two groups in basal conditions of the 2^nd^ year showed: for VAS, P = 0.60; for PPTs in TePs, P = 0.08; for EPTs in skin, P = 0.44; for EPTs in subcutis, P = 0.61; for EPTs in muscle, P = 0.54; for PPTs in muscle, P = 0.82.

#### Postoperative pain

No complications occurred with surgery in the 20 patients scheduled for cholecystectomy; laparoscopy was successfully carried out in all of them.

VAS scores for pain perceived at abdominal surgery site in the postoperative period are reported in Fig 4B. These were significantly higher with respect to non-fibromyalgia patients undergoing cholecystectomy during the first year [sGb+Cholec] at all evaluation times and not significantly different from scores of fibromyalgia patients who underwent laparoscopic surgery during the first year [FMS+sGb+Cholec].

#### Evolution of pain parameters during the second year

**Fig 11:**
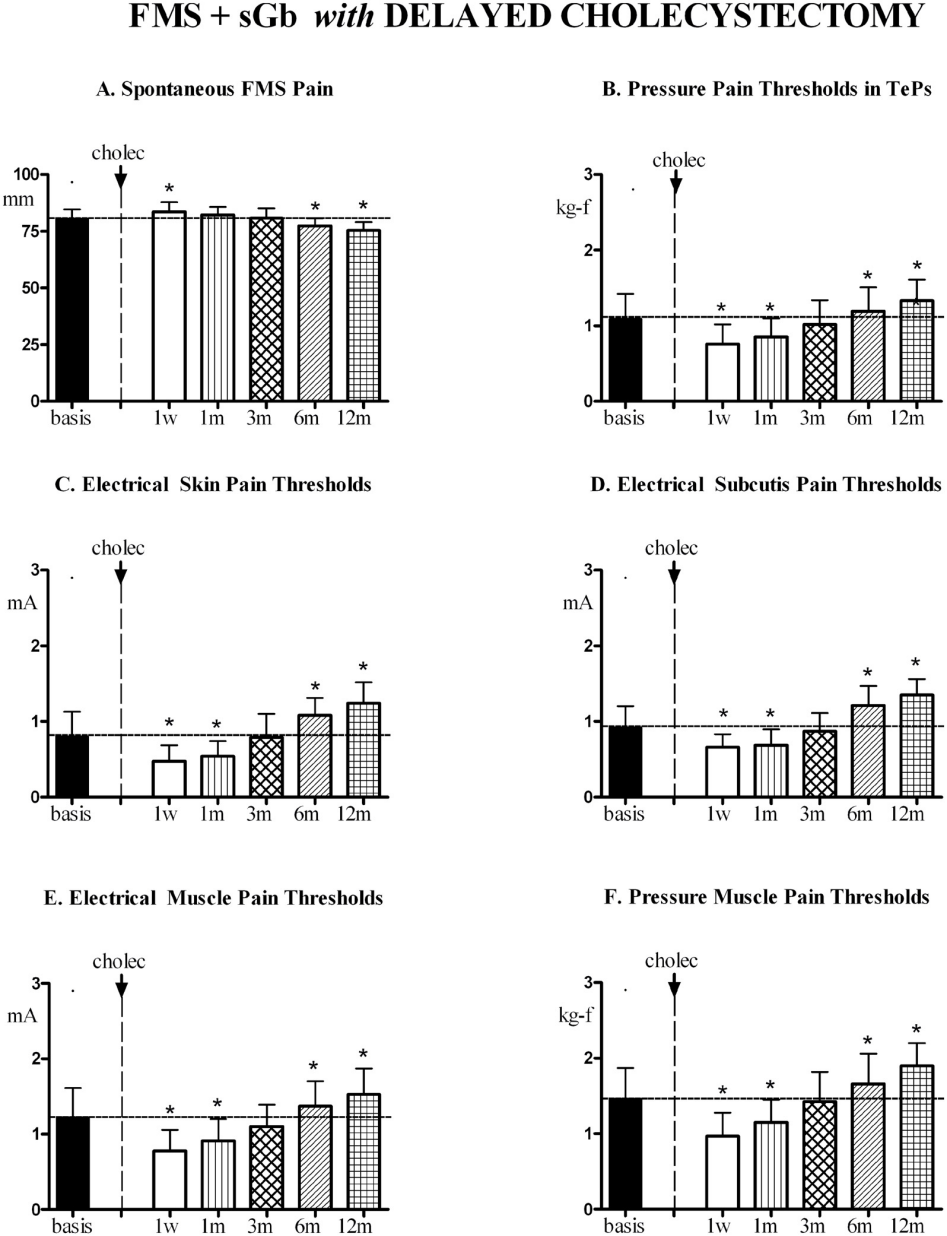
FMS symptoms and pain sensitivity in [FMS+sGb with delayed Cholec]. Patients with fibromyalgia (FMS) plus symptomatic gallbladder calculosis (sGb) subjected to cholecystectomy during the second year of study (delayed cholecystectomy) (n = 20, Means ± SD). ANOVA for repeated measures: for VAS, (P<0.0001; F = 33.608); for PPTs in TePs (P<0.0001; F = 89.276, for EPTs in skin (P<0.0001; F = 25.000), for EPTs in subcutis (P<0.0001; F = 52.969), for EPTs in muscle ((p<0.0001; F = 69.778), for PPTs in muscle (p<0.0001; F = 76.896). The asterisk over SD bars indicates a significant difference with respect to basal values.

In [FMS+sGb with delayed Cholec] the evolution of FMS parameters and pain sensitivity in control areas during this second year is reported in Fig 11.

A significant trend for variation was present for all parameters. FMS pain intensity significantly increased 1 week after cholecystectomy, to return to preoperative values at 1 and 3 months and then significantly decrease at 6 and 12 months (A). All thresholds significantly decreased 1 week and 1 month after cholecystectomy, to return to pre-operative values at 3 months and then significantly increase at 6 and 12 months (B-F).

**Fig 12:**
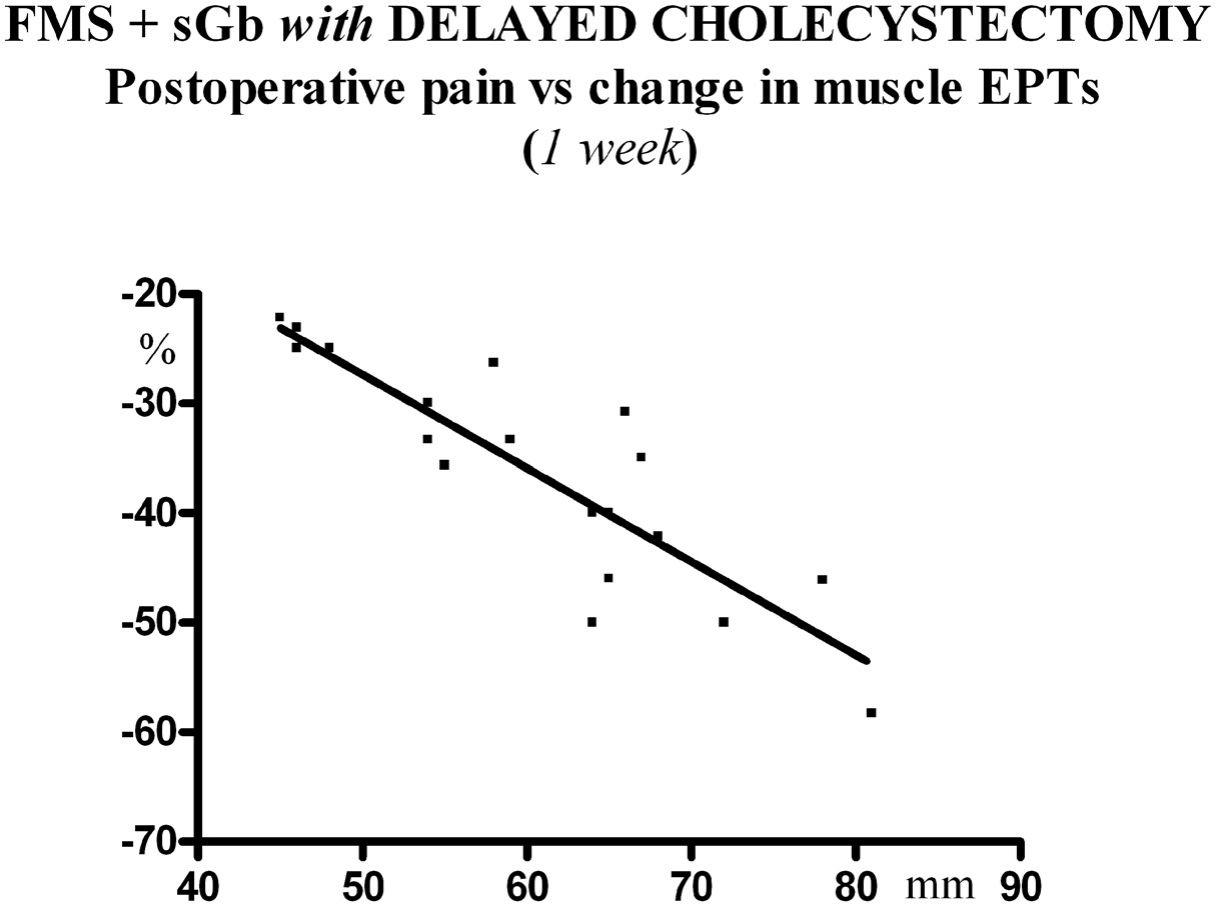
Patients [FMS+sGb with delayed Cholec] (n = 20). Linear correlation between peak postoperative pain and change (decrease) in electrical pain thresholds at control sites at 1^st^ week.

A significant inverse linear correlation was found between the peak postoperative pain experienced at the operation site during the first week after laparoscopy and the change (decrease) in electrical pain thresholds at control sites 1 week after surgery (Y = -0.8520 + 15.23; (r) = -0.8731; p<0.0001) (Fig 12).

All other explored correlations were not significant, as follows:

Postoperative pain vs change in FMS pain: at 1 week (P = 0.7218; (r) = 0.08494), at 1 month (P = 0.6242; (r) = -0.1167), at 3 months (P = 0.2870; (r) = 0.2504), at 6 months (P = 0.4411; (r) = 0.1826), at 12 months (P = 0.6644; (r) = 0.1034);

Postoperative pain vs change in PPTs in TePs: at 1 week (P = 0.8579; (r) = -0.04277), at 1 month (P = 0.8682; (r) = -0.03965), at 3 months (P = 0.6754; (r) = -0.09983), at 6 months (P = 0.3824; (r) = -0.2065), at 12 months (P = 0.3977; (r) = 0.2001);

Postoperative pain vs change in skin EPTs: at 1 week (P = 0.6274; (r) = -0.1156), at 1 month (P = 0.1637; (r) = 0.3238), at 3 months (P = 0.3414, (r) = -0.2245), at 6 months (P = 0.2845; (r) = -0.2516), at 12 months (P = 0.9905;(r) = 0.002855);

Postoperative pain vs change in subcutis EPTs: at 1 week (P = 0.2431;(r) = -0.2736), at 1 month (P = 0.2383; (r) = -0.2763), at 3 months (P = 0.2938; (r) = -0.2470), at 6 months (P = 0.1302; (r) = -0.3501), at 12 months (P = 0.6274; (r) = -0.1156);

Postoperative pain vs change in muscle EPTs: at 1 month (P = 0.0569; (r) = 0.4324), at 3 months (P = 0.0512; (r) = 0.4417), at 6 months (P = 0.3670;(r) = 0.2131), at 12 months (P = 0.2345; (r) = 0.2785);

Postoperative pain vs change in muscle PPTs: at 1 week (P = 0.8073; (r) = -0.05825), at 1 month (P = 0.5048; (r) = -0.1584), at 3 months (P = 0.3744, (r) = -0.2099), at 6 months (P = 0.7350, (r) = 0.08075), at 12 months (P = 0.5447; (r) = 0.1440).

**Fig 13:**
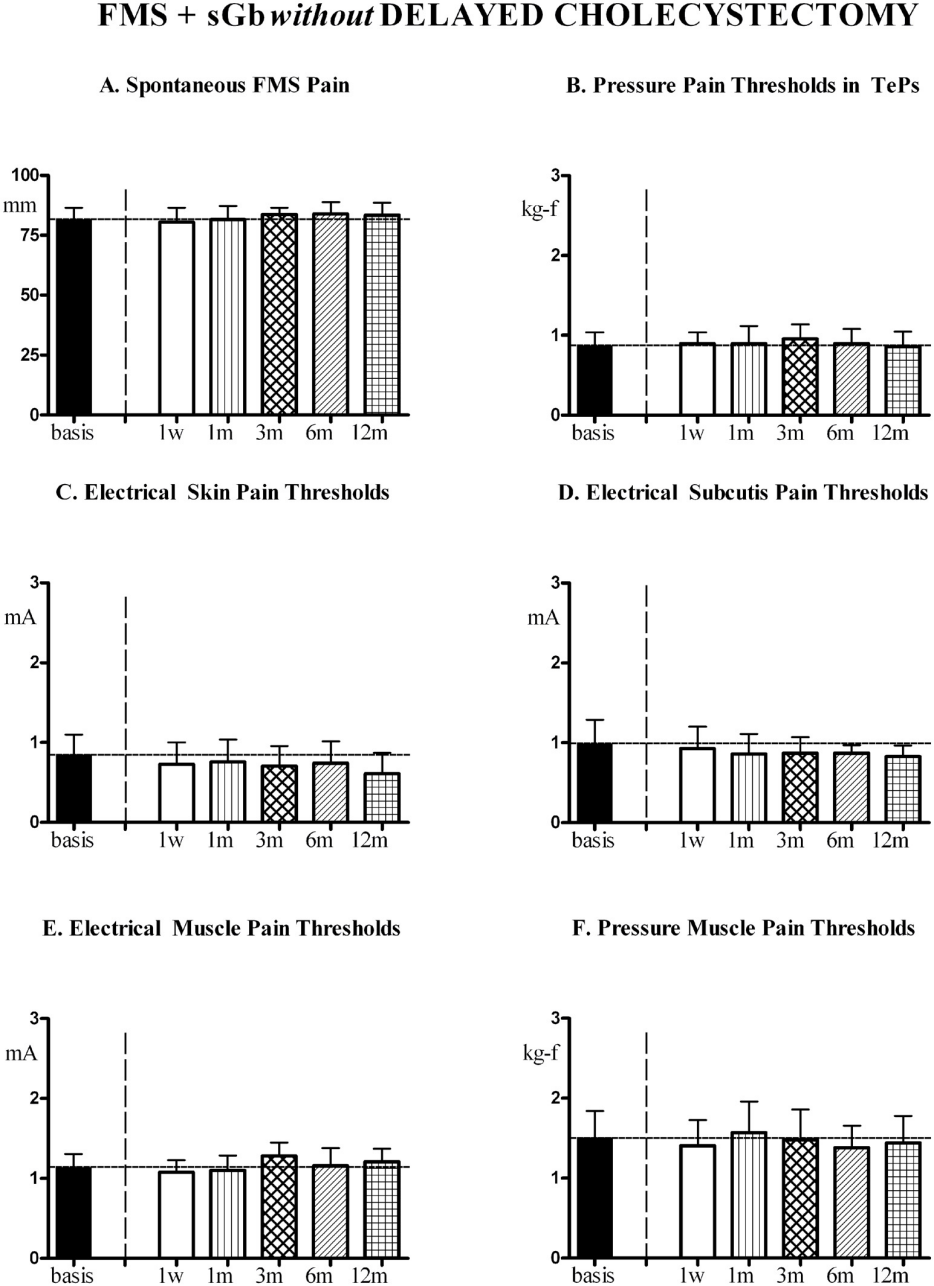
FMS symptoms and pain sensitivity in [FMS+sGb without delayed Cholec]. Patients with fibromyalgia (FMS) plus symptomatic gallbladder calculosis (sGb), not subjected to cholecystectomy (n = 7, Means ± SD) for a period of 2 years. Evaluation performed during the 2^nd^ year at comparable time points as patients of Fig 11. No significant trend for all parameters. ANOVA for repeated measures: for VAS, (P = 0.1098; F = 1.984), for PPTs in TePs (P = 0.6121; F = 0.7220, for EPTs in skin (P = 0.4864; F = 0.9122), for EPTs in subcutis (P = 0.5140, F = 0.8681), for EPTs in muscle (P = 0.1644; F = 1.703), for PPTs in muscle (P = 0.5311; F = 0.8414).

In [FMS+sGb without delayed Cholec] the profile of FMS symptoms and pain sensitivity in control areas during this second year, evaluated at the same time points as patients subjected to cholecystectomy, is reported in Fig 13. No significant trend for variation was found for any of the evaluated parameters.

## Discussion and Conclusions

This study on fibromyalgia patients with and without gallbladder calculosis comorbidity, subjected or not to laparoscopic cholecystectomy, has highlighted a number of outcomes in relation to spontaneous FMS pain and pain sensitivity at somatic level.

Firstly, all fibromyalgia patients are confirmed as presenting generalized hypersensitivity to pain stimuli in all three tissues of the body wall, as shown by significantly decreased pain thresholds to more than one modality of stimulus (pressure and electrical) with respect to non-fibromyalgia patients [8,10]. Secondly, in fibromyalgia with comorbid calculosis, biliary colics appear as an exacerbating factor for FMS pain and hypersensitivity. In fact, while in basal conditions FMS patients with asymptomatic calculosis do not substantially differ from FMS patients without gallbladder pathology, significantly higher levels of spontaneous FMS pain, TeP tenderness and generalized hyperalgesia are observed in FMS plus symptomatic calculosis vs the previous two groups. Furthermore, the extent of FMS symptoms and hypersensitivity is directly correlated with that of the pain from calculosis, i.e, the higher the number of previously experienced colics, the higher the level of spontaneous diffuse FMS pain, tenderness at tender point sites and diffuse muscle hyperalgesia, as testified by decreased electrical and pressure pain thresholds at somatic level.

This outcome of enhanced FMS pain/hypersensitivity due to the biliary colics is very similar to what was previously observed for other peripheral pain comorbidities in FMS patients, such as myofascial pain syndromes (from trigger points) or painful joints, which were also shown to worsen FMS symptoms, and whose effective local treatment significantly improved them [7,12,30]. The effect of the so-called “peripheral pain generators” in fibromyalgia is an increasingly acknowledged aspect of FMS pathophysiology and, as a consequence, of the therapeutic approach to the syndrome [11].

Laparoscopic cholecystectomy in all of our patients produced a complete and longlasting resolution of biliary symptoms. This outcome differs from a number of previous studies showing several persisting pain and non-pain consequences of the intervention, including symptoms from gastroparesis, significantly more frequent in patients with cholecystectomy than in those without, i.e., early satiety, postprandial fullness, nausea, vomiting, and abdominal pain [31]. However, our outcome may be due to the strict selection criteria for patients in the study: those with any preoperative risk factor for conversion were, in fact, excluded. Our patients were thus free from many of the comorbidities which may favour postoperative pain complications (i.e., ischemic heart disease, uncontrolled arterial hypertension, liver pathologies, chronic obstructive pulmonary disease; severe obesity, diabetes, previous pancreatitis, ultrasound signs of acute cholecystitis, previous upper abdominal surgery).

A third outcome of our study is that laparoscopic cholecystectomy in FMS plus symptomatic gallbladder calculosis, while producing a complete and longlasting resolution of biliary symptoms, instead determined a relatively transient worsening of FMS symptoms and hypersensitivity, at 1 week and 1 month, compared to preoperative levels. At 3 months, all FMS symptoms had returned to pre-surgery values and then significantly improved in the long term, at 6 and 12 months.

This time evolution of FMS symptoms was substantially different from that of FMS patients with or without gallbladder calculosis, who, although not undergoing cholecystectomy, were assessed at comparable time points over a period of 1 year. These patients, in fact, showed a substantial stable pattern of all parameters over time, with only a slight worsening at the end of the year for the group with FMS plus symptomatic calculosis. The post-cholecystectomy time evolution of general pain sensitivity in FMS+symptomatic calculosis also differed from that of patients with gallbladder calculosis without fibromyalgia. In the latter group, in fact, thresholds in all somatic tissues did not change significantly from preoperative values, confirming results of previous studies showing no impact of this intervention in non-fibromyalgic patients on pain sensitivity in areas outside that of pain referral from the gallbladder (quadriceps)[23].

A further aspect of our study regards pain at the surgery site during the first week after laparoscopy. The levels of this pain in non-fibromyalgia patients were of relatively moderate intensity and comparable in extent and time evolution to those already reported in previous studies [27]. However, they were significantly higher in fibromyalgia than non-fibromyalgia patients, in line with the higher reactivity to painful/traumatic events typical of FMS. Furthermore, the peak postoperative pain at the surgery site in FMS correlated directly with the increased hyperalgesia at muscle level found 1 week and 1 month after the operation. Although the influence of other factors, such as postoperative levels of anxiety, cannot be entirely excluded, this result suggests that in a first phase after laparoscopy the afferent barrage from the surgery site enhances the level of central sensitization in FMS, which is reflected in the higher FMS symptoms found up to the first month. In the long run, instead, from the third month onwards, it would seem that the positive effect of abolishing pain from the gallbladder prevails, i.e., eliminating the source of repeated visceral noxious inputs would produce some degree of desensitization, with consequent stable improvement of FMS symptoms.

The effects of cholecystectomy on central sensitization have been explored in non-fibromyalgic subjects in previous studies. Stawowy et al [32] applied quantitative sensory testing in the referred pain area from the gallbladder (upper abdominal quadrant) and in a control area on the contralateral side of the abdomen before and 4–12 weeks after surgery, while Kjaer et al [33] performed a similar study evaluating the somatosensory changes 2–7 years after laparoscopic cholecystectomy. In both cases the initial hypersensitivity of the referred area returned to normal in the follow-up period, also in patients who developed postcholecystectomy syndrome. Therefore, the neuroplastic changes seen before surgery seem to disappear after successful surgery, at least in non-FMS patients. The minimal invasive procedures are likely to be responsible for the findings [34]. As already reported above, also a previous study by our group, in patients with symptomatic gallbladder calculosis without fibromyalgia, demonstrated no effect of the surgery/laparoscopic procedure on the general pain sensitivity to pain outside the area of pain referral (quadriceps) 1 month after the operation [23]. In the present study we did not evaluate the area of pain referral from the gallbladder, as we did previously in studies focused on visceral pain [22,35,36] since our aim here was primarily to assess the effects of cholecystectomy on the generalized pain hypersensitivity at somatic level typical of FMS, rather than compare the level of referred visceral hypersensitivity in fibromyalgia vs non fibromyalgia patients. It would be of interest, however, to also explore this aspect in future studies.

In conclusion, our results show that laparoscopic cholecystectomy for gallbladder calculosis in fibromyalgia produces only a transitory worsening of FMS symptoms in the postoperative period, which is largely compensated by the long-term outcome of desensitization due to gallbladder removal. This study provides new insights into the role of visceral pain comorbidities for central pain in FMS, helping the planning of adequate therapy of these comorbidities as a tool to also control FMS symptoms.

## Supporting Information

S1 Table Biliary Colics. Number of biliary colics experienced by patients with symptomatic gallbladder calculosis. Raw data.
(ISD)

S2 Table (A)VAS of FMS pain. Intensity of fibromyalgia pain in FMS+sGb+Cholec, FMS+aGb, FMS and FMS+sGb patients. Raw data.
(ISD)

S3 Table (B)VAS of FMS pain. Intensity of fibromyalgia pain in FMS+sGb with delayed Cholec and FMS+sGb without delayed Cholec patients. Raw data.
(ISD)

S4 Table (A)PPTs in TePs. Pressure pain thresholds in tender points in FMS+sGb+Cholec, FMS+aGb, FMS and FMS+sGb patients. Raw data.
(ISD)

S5 Table (B)PPTs in TePs. Pressure pain thresholds in tender points in FMS+sGb with delayed Cholec and FMS+sGb without delayed Cholec patients. Raw data.
(ISD)

S6 Table (A)EPTs in skin and subcutis. Electrical skin and subcutis pain thresholds in FMS+sGb+Cholec and FMS patients. Raw data.
(ISD)

S7 Table (B)EPTs in skin and subcutis. Electrical skin and subcutis pain thresholds in FMS+aGb and FMS+sGb patients. Raw data.
(ISD)

S8 Table (C)EPTs in skin and subcutis. Electrical skin and subcutis pain thresholds in FMS+sGb with delayed Cholec and FMS+sGb without delayed Cholec patients. Raw data.
(ISD)

S9 Table EPTs in muscle. Electrical muscle pain thresholds in FMS+sGb+Cholec, FMS+aGb, FMS and FMS+sGb patients. Raw data.
(ISD)

S10 Table PPTs in muscle. Pressure pain thresholds in muscle in FMS+sGb+Cholec, FMS+aGb, FMS and FMS+sGb patients. Raw data.
(ISD)

S11 Table EPTs and PPTs in muscle. Electrical and pressure pain thresholds in muscle in FMS+sGb with delayed Cholec and FMS+sGb without delayed Cholec patients. Raw data.
(ISD)

S12 Table EPTs and PPTs in skin, subcutis and muscle. Electrical and pressure pain thresholds in the three tissues of the body wall in sGb+Cholec patients. Raw data.
(ISD)

S13 Table VAS of postoperative pain. Intensity of pain at the surgical site experienced by patients subjected to laparoscopic cholecystectomy (during 1 week after surgery). Raw data.
(ISD)

## Abbreviations

aGb: asymptomatic gallbladder calculosis
AUs: abdominal ultrasounds
Cholec: cholecystectomy
EPTs: electrical pain thresholds
FMS: fibromyalgia syndrome
NSAIDs: Non-Steroidal Antiinflammatory Drugs
PPTs: pressure pain thresholds
sGb: symptomatic gallbladder calculosis
TePs: tender points
